# Identification of potentially causative drugs associated with hypotension: A scoping review

**DOI:** 10.1002/ardp.202400564

**Published:** 2024-11-28

**Authors:** Nurunnisa Sari, Ulrich Jaehde, Anna Maria Wermund

**Affiliations:** ^1^ Institute for Medical Information Processing, Biometry and Epidemiology ‐ IBE LMU Munich Munich Germany; ^2^ Pettenkofer School of Public Health Munich Munich Germany; ^3^ Department of Clinical Pharmacy, Institute of Pharmacy University of Bonn Bonn Germany

**Keywords:** adverse drug events (ADEs), antihypertensive drugs, drug‐induced hypotension, pharmacovigilance, systematic literature search

## Abstract

Drug‐induced hypotension can be harmful and may lead to hospital admissions. The occurrence of hypotension during drug therapy is preventable through increased awareness. This scoping review aimed to provide a comprehensive overview of antihypertensive and nonantihypertensive drugs associated with hypotension in adults. A systematic literature search was conducted using MEDLINE, Embase and Cochrane Library, focusing on studies from January 2013 to May 2023. Search terms were developed to capture key concepts related to hypotension and adverse drug events in adults while excluding terms related to allergic reactions, phytotherapy and studies involving paediatric, pregnant or animal populations. The eligibility criteria included a wide range of study types evaluating hypotension as an adverse drug event across all healthcare settings. Relevant information was extracted from the included studies, while identified drugs associated with hypotension were categorised into drug classes. The review was reported using the Preferred Reporting Items for Systematic Reviews and Meta‐Analyses Extension for Scoping Reviews checklist. In 97 eligible studies, we identified 26 antihypertensive drugs grouped into nine different antihypertensive classes and 158 other drugs grouped into 22 other drug classes. Common antihypertensive classes were angiotensin‐converting enzyme inhibitors, beta blockers and diuretics. Frequently reported nonantihypertensive classes were neuroleptics, alpha‐1 blockers for benign prostatic hyperplasia, benzodiazepines, opioids and antidepressants. The results highlight the importance of healthcare professionals being aware of nonantihypertensive drugs that can cause hypotension. This review provides a basis for future systematic reviews to explore dose‐dependence, drug–drug interactions and confounding factors.

## Graphical abstract

Antihypertensives are just the tip of the iceberg of drugs that cause hypotension. This scoping review examines antihypertensive and nonantihypertensive drugs associated with hypotension, analysing 97 studies from 2013 to 2023. It examines the frequency of reporting and corresponding levels of evidence and evaluates the results taking into account mechanisms of action, potential confounders, healthcare settings and prescription frequency.

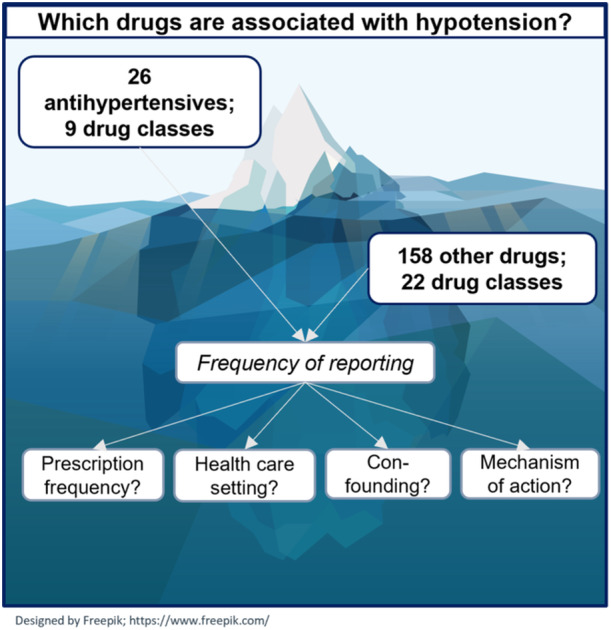

## 1 INTRODUCTION

### 1.1 Background and rationale

Hypotension, defined as a blood pressure below 90/60 mmHg, can be harmful to the patient.^[^ ^1^ ^]^ Due to its potential to cause organ hypoperfusion, it can lead to serious health consequences such as syncope, falls and cognitive impairment.^[^ ^1^, ^2^, ^3^, ^4^ ^]^ The increasing number of hospital admissions due to hypotension is a major concern. A study of hospital admission data between 1999 and 2020 showed that hypotension‐related admission rates increased by 149% in Wales, 168% in Australia and 398% in England, highlighting the importance of investigating the causes of hypotension.^[^ ^5^ ^]^

A considerable proportion of hypotension‐related hospital admissions are drug‐related. In the study mentioned above, drugs accounted for 7.6% of all hypotension‐related admissions in England, 10% in Wales and 13.5% in Australia.^[^ ^5^ ^]^ A cross‐sectional study identified hypotension and falls as the most common adverse drug events (ADEs) associated with hospital admissions, accounting for 24.1% of all admissions related to ADEs.^[^ ^6^ ^]^ Similarly, in a cohort study of hospitalised elderly patients, hypotension was the most common ADE, representing 19.5% of all ADEs.^[^ ^7^ ^]^ In another cross‐sectional study, hypotension was one of the most commonly observed preventable ADEs (12% of preventable ADEs), underlining the importance of drugs as preventable causes of hypotension.^[^ ^8^ ^]^ Knowledge of these potentially causative drugs for hypotension can lead to more tailored and proactive management strategies (e.g., risk models, clinical decision support systems) to minimise the risks associated with drug‐induced hypotension and increase awareness among healthcare professionals. Previous reviews on drug‐induced hypotension have identified potential causative drugs, such as alpha‐1 blockers and neuroleptics, but a comprehensive overview of the evidence base is still lacking. Many reviews have focused on orthostatic hypotension (OH), neglecting the broader issue of generalised hypotension.^[^ ^9^, ^10^, ^11^, ^12^, ^13^ ^]^ In addition, most of these reviews are narrative and lack a systematic and comprehensive approach.^[^ ^9^, ^11^, ^12^, ^13^ ^]^ A recently published systematic review (SR) focused only on OH and limited its search to randomised controlled trials (RCTs).^[^ ^10^ ^]^ Thus, a substantial part of the research landscape remains to be explored. To fill this gap, we used the scoping review (ScR) methodology to provide a comprehensive overview of the available evidence on potentially causative drugs. By examining a broader range of study designs, this method allows a more comprehensive exploration of hypotension‐inducing drugs and can guide future research by highlighting evidence gaps, such as rarely represented drugs.

### 1.2 Objectives and review question

The aim of this ScR was to identify potential causative drugs for hypotension in adults and to construct a comprehensive evidence map that is not restricted to specific study types or healthcare settings. Therefore, the central research question that guided our review was: ‘What drugs have been associated with hypotension in adults and what type of evidence supports these associations?’

## 2 METHODS

We conducted an ScR using the Joanna Briggs Institute (JBI) methodology for ScRs and following the Preferred Reporting Items for Systematic Reviews and Meta‐Analyses Extension for Scoping Reviews (PRISMA‐ScR) guidelines.^[^ ^14^, ^15^, ^16^ ^]^ Before the literature search, we developed an ScR protocol with an adaptable systematic search strategy to identify drugs potentially responsible for any ADE, regardless of the study design or clinical setting. This protocol is available in Supporting Information: Supplement S1.

### 2.1 Development of search strategy

First, previous studies on ADEs were identified to collect relevant keywords and phrases for ADEs, from which an initial search strategy for potentially causative drugs was developed. This general ADE search strategy was then combined with the hypotension search strategy to limit our review to the specific ADE in focus. The inclusion and exclusion criteria and associated search terms were refined after an initial search in MEDLINE and screening of the first 100 results. The search strategy included keywords (MeSH or Emtree) and text words. The detailed search strategy and search dates are presented in Supporting Information: Supplement S2.

### 2.2 Data sources and eligibility criteria

A comprehensive literature search was conducted in Embase via OVID, MEDLINE via PubMed and the Cochrane Library, covering publications from 1 January 2013 to 25 May 2023 and including all studies that evaluated hypotension as an ADE in all healthcare settings. To ensure a comprehensive overview, various types of studies were included, such as reviews of any kind, meta‐analyses (MAs), observational studies, RCTs and noncontrolled studies. All publications mentioning hypotension in the context of drug therapy were included. Studies were eligible if they were published in English or German, regardless of their geographical origin. All inclusion and exclusion criteria are listed in Table 1. In addition to the primary and secondary publications from the databases, reports of a tertiary publication by Anne Lee were also considered.^[^ ^17^ ^]^

**Table 1:** Study inclusion and exclusion criteria.

	Inclusion criteria	Exclusion criteria
Population		− P1: Age <18 years − P2: Pregnancy
Event	− Hypotension as a side effect of drug therapy	− E1: Hypotension as a symptom of another ADE (e.g., anaphylaxis, capillary leak syndrome, hypersensitivity, infusion‐related reactions) − E2: Drug–drug interactions or combination therapies (except for fixed‐dose drug combinations, including e.g., tadalafil + tamsulosin, buprenorphine + naloxone, sacubitril + valsartan) − E3: Drug toxicity due to overdose, overuse, poisoning, medication errors, drug abuse − E4: Medical devices, excipients − E5: Herbal medicinal products, traditional Chinese medicine − E6: Hypotension not as ADE (e.g., drug treatment of hypotension) − E7: No difference from placebo
Context	− Any care setting	− C0: Poison centres
Study	− Phase II/III–IV clinical trials − All types of reviews (e.g., rapid, living, scoping, systematic) − RCTs − Noncomparative/noncontrolled experimental studies (single‐arm studies) − Observational studies (case–control, cross‐sectional, cohort studies); prospective and retrospective designs − Retrospective analysis of pharmacovigilance database data	− S1: No original results for drugs that cause hypotension, except for narrative reviews − S2: Preclinical studies; animal studies; phase I‐II clinical trials − S3: Case reports and case series (except for retrospective analyses of pharmacovigilance databases) − S4: No association of hypotension with a certain drug − S5: Only registration in clinical trial databases (e.g., clinicaltrials.gov) without results − S6: Language other than English or German − S7: Conference abstracts

Abbreviations: ADE, adverse drug event; RCTs, randomised controlled trials.

We excluded hypotension due to allergic reactions to focus on direct pharmacological effects. Studies involving medication errors, poisoning or drug abuse were not included to keep the focus on standard therapeutic contexts in accordance with the Summary of Product Characteristics (SmPCs). Phytotherapeutic drugs were excluded due to their variable composition and quality. Studies involving drugs with no differential hypotensive effect compared with placebo, or those not specifying the underlying hypotensive drug or drug group, were excluded. In comparative studies, all drugs inducing hypotension were included without hierarchical grading of their effects, thereby ensuring a qualitative rather than quantitative analysis of drug‐induced hypotension. Conference abstracts were excluded because they did not contain the detailed data required for an in‐depth analysis.

### 2.3 Study selection

The online SR software Rayyan was used to facilitate the removal of duplicates and literature screening. Two reviewers (N. S. and A. M. W.) independently screened all identified titles and abstracts to determine their eligibility for inclusion. Subsequently, the full texts of the eligible studies were evaluated. In both steps, if disagreements appeared, the reviewers discussed the reasons for inclusion.

### 2.4 Data extraction

An extraction template was prepared using Microsoft Excel^TM^ to systematically document key information from each study. The extracted data included (I) publication title, (II) first author, (III) year of publication, (IV) study type, (V) healthcare setting and (VI) population characteristics (such as country, age and special conditions). When possible, each study was assigned a level of evidence according to the Oxford Centre for Evidence‐Based Medicine.^[^ ^18^ ^]^ The following study types were not included in this evidence scale and were therefore added by the authors: SRs without MA, cross‐sectional studies, other observational studies and nonrandomised interventional studies. The category ‘other observational studies’ encompasses observational studies that could not be classified into a specific observational study type due to a deficiency of information in the publication. The category ‘non‐randomised interventional studies’ covers interventional studies that were performed without randomisation of the cohort. The category ‘case series’ in our research refers to studies which carry out retrospective evaluations of data extracted from pharmacovigilance databases. All of the study types included and their corresponding levels of evidence are listed in Table 2. The term ‘high level of evidence’ pertains to SRs of RCTs with MA or to the RCTs themselves. N. S. extracted the relevant information from the included publications, while A. M. W. carried out a sample verification of this information.

**Table 2:** Levels of evidence, graded/listed by decreasing strength of evidence.

Level of evidence according to the Oxford Centre for EbM	Study type
1a	Systematic reviews of randomised controlled studies with meta‐analysis
1b	Randomised controlled studies
[a]	Systematic reviews without meta‐analysis
2b	Cohort studies
3b	Case–control studies
[a]	Cross‐sectional studies
[a]	Other observational studies
[a]	Nonrandomised interventional studies
4	Case series (in our case pharmacovigilance database analyses)
5	Expert opinions (in our case mainly narrative reviews)

*Note*: [a] no representation in Oxford Centre for EbM.

Abbreviation: EbM, evidence‐based medicine.

A separate sheet was used to extract drugs or drug classes associated with hypotension. The extracted drugs or drug classes were allocated to the evidence levels of the studies that mentioned them. The extracted drugs were grouped into superordinate drug classes according to their mechanism of action. These superordinate drug classes were either named in the literature or defined by the researchers based on the mechanism of action. Supporting Information: Supplement S3 clarifies which drug classes were reported in the existing literature by name and which ones were newly defined in this review. For larger drug classes, such as antidepressants, analgesics and neuroleptics, further differentiation was made by separating them into underlying subclasses. The drug classes were categorised into two groups: ‘antihypertensives’ and ‘other drug classes’. The term ‘antihypertensives’ refers to drugs indicated for the treatment of hypertension, irrespective of additional indications such as renal disease or heart failure. Drugs that could not be assigned to a drug class were grouped under the term ‘unclassified other drugs’. Additionally, we noted whether these drugs or drug classes were reported to be associated with OH.

### 2.5 Data visualisation

To visualise the number and distribution of publications across different levels of evidence for each drug class or drug, three evidence diagrams with stacked bar graphs were generated for antihypertensives, other drug classes and other unclassified drugs.

To construct the evidence diagrams, we used the extracted data from Supporting Information S1: Supplement S3, which lists the sources that mentioned each individual drug or a particular subclass or class of drugs. We used a hierarchical aggregation approach to present this information comprehensively for each drug class. In particular, we assigned mentions of individual drugs belonging to subclasses or drug classes to their respective classes. For example, the bar graph for tricyclic antidepressants (TCAs) aggregates references to all individual drugs within this subclass. Similarly, the bar graph for the broader class of antidepressants combines all references that mention an individual antidepressant or an antidepressant subclass.

References that mentioned alpha‐1 blockers in general were counted for both classes: alpha‐1 blockers used for benign prostatic hyperplasia (BPH) and alpha‐1 blockers used as antihypertensives.

## 3 RESULTS

### 3.1 Literature search

**Figure 1:**
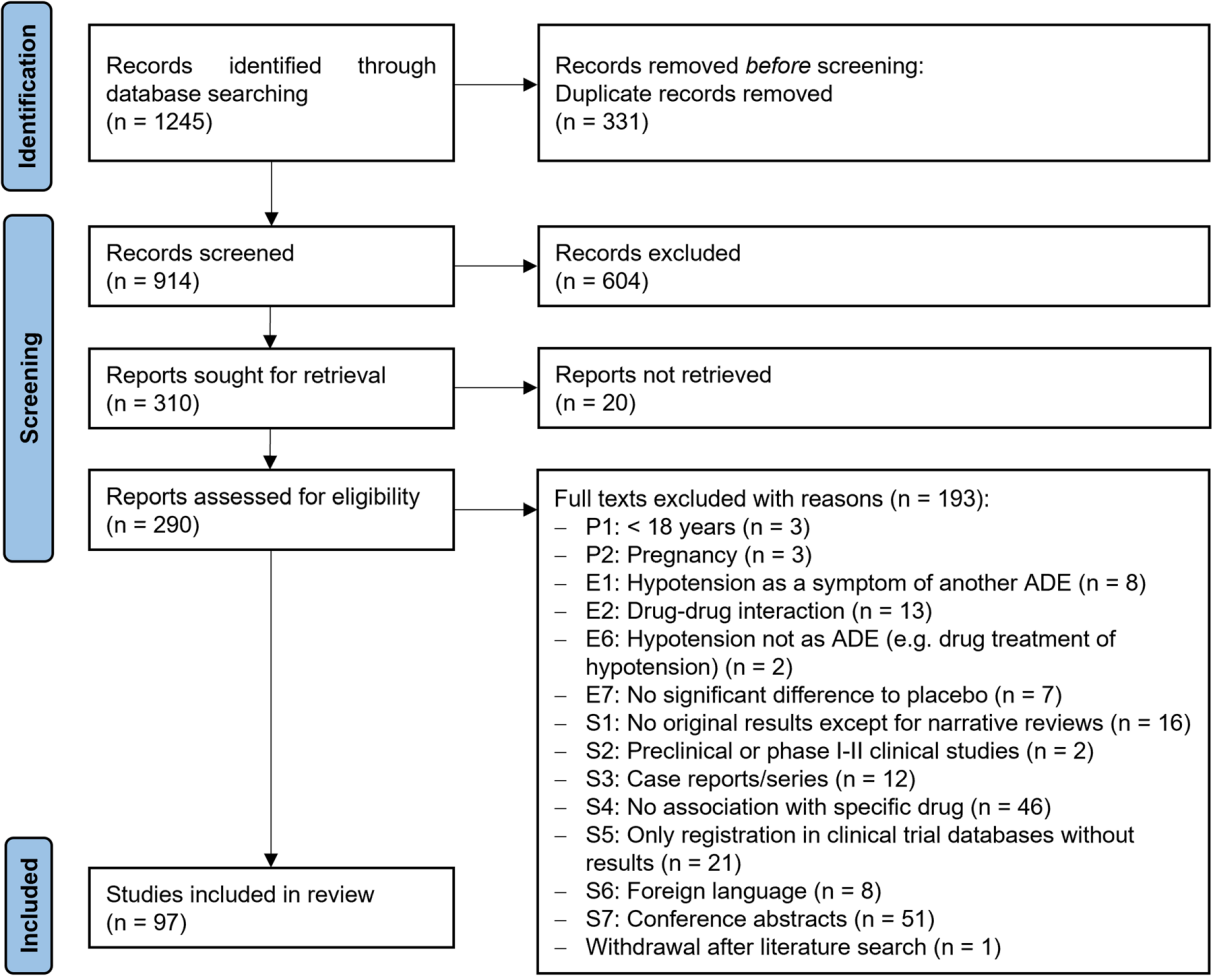
PRISMA Flowchart according to the PRISMA statement.^[^ ^16^ ^]^ ADE, adverse drug event.

Our literature search retrieved 1245 citations. The screening of titles and abstracts resulted in the selection of 290 articles for full‐text review, as 20 of the 310 titles included by abstract were not accessible as a full text. The screening process of titles and abstracts resulted in 78 cases of disagreement regarding inclusion or exclusion. After discussion, it was decided to include 60 of the articles. During the full‐text screening, disagreements occurred for five articles. Following further review, three of these were ultimately included in the final selection. Of the 98 articles that initially met our inclusion criteria, one was retracted after the completion of our literature search. Consequently, the results of this article were excluded from our analysis. Therefore, our review included a total of 97 publications, as shown in Figure 1.

### 3.2 Study characteristics

Supporting Information S1: Supplement S4 provides an overview of the 97 studies included in our review.^[^ ^7^, ^10^, ^11^, ^19^, ^20^, ^21^, ^22^, ^23^, ^24^, ^25^, ^26^, ^27^, ^28^, ^29^, ^30^, ^31^, ^32^, ^33^, ^34^, ^35^, ^36^, ^37^, ^38^, ^39^, ^40^, ^41^, ^42^, ^43^, ^44^, ^45^, ^46^, ^47^, ^48^, ^49^, ^50^, ^51^, ^52^, ^53^, ^54^, ^55^, ^56^, ^57^, ^58^, ^59^, ^60^, ^61^, ^62^, ^63^, ^64^, ^65^, ^66^, ^67^, ^68^, ^69^, ^70^, ^71^, ^72^, ^73^, ^74^, ^75^, ^76^, ^77^, ^78^, ^79^, ^80^, ^81^, ^82^, ^83^, ^84^, ^85^, ^86^, ^87^, ^88^, ^89^, ^90^, ^91^, ^92^, ^93^, ^94^, ^95^, ^96^, ^97^, ^98^, ^99^, ^100^, ^101^, ^102^, ^103^, ^104^, ^105^, ^106^, ^107^, ^108^, ^109^, ^110^, ^111^, ^112^ ^]^ Of these studies, less than 10% (*n* = 7) were SRs of RCTs including MAs.^[^ ^10^, ^22^, ^24^, ^36^, ^56^, ^81^, ^88^ ^]^ Most of the included studies were observational, accounting for 36% of the studies.^[^ ^7^, ^19^, ^20^, ^23^, ^27^, ^31^, ^34^, ^35^, ^40^, ^43^, ^47^, ^48^, ^53^, ^54^, ^64^, ^65^, ^76^, ^77^, ^78^, ^82^, ^84^, ^86^, ^87^, ^95^, ^96^, ^98^, ^99^, ^100^, ^102^, ^103^, ^107^, ^109^, ^110^, ^111^, ^112^ ^]^ More than 20% were interventional studies, consisting of 15 RCTs and six nonrandomised trials (*n* = 6).^[^ ^21^, ^38^, ^41^, ^42^, ^44^, ^45^, ^46^, ^50^, ^55^, ^60^, ^69^, ^70^, ^71^, ^74^, ^79^, ^80^, ^83^, ^85^, ^91^, ^94^, ^97^ ^]^ Narrative reviews comprised 21% of the studies.^[^ ^11^, ^29^, ^30^, ^32^, ^37^, ^49^, ^51^, ^57^, ^59^, ^61^, ^62^, ^63^, ^68^, ^72^, ^73^, ^90^, ^92^, ^105^, ^106^, ^108^ ^]^ A total of 9% of eligible publications were retrospective analyses of data extracted from pharmacovigilance databases, including the World Health Organisation (WHO) Vigibase, the Food and Drug Administration (FDA) Adverse Event Reporting System (FAERS) and the Korean Adverse Event Reporting System (KAERS).^[^ ^25^, ^26^, ^28^, ^39^, ^52^, ^58^, ^66^, ^67^, ^75^ ^]^ One of the identified publications was an analysis of Summaries of Product Characteristics (SmPCs).^[^ ^89^ ^]^

The study centres in this review were predominantly located across Asia, accounting for 22% of the included studies.^[^ ^19^, ^21^, ^27^, ^34^, ^35^, ^41^, ^44^, ^45^, ^47^, ^60^, ^65^, ^67^, ^77^, ^78^, ^79^, ^80^, ^83^, ^85^, ^100^, ^110^, ^112^ ^]^ A total of 16% were conducted in North America, while 6% of the studies were carried out in European countries.^[^ ^20^, ^25^, ^28^, ^39^, ^40^, ^43^, ^46^, ^50^, ^52^, ^53^, ^54^, ^64^, ^82^, ^84^, ^86^, ^87^, ^95^, ^98^, ^99^, ^102^, ^103^, ^107^, ^111^ ^]^ Only a small proportion of 5% of study centres were located in South America, North Africa or Australia.^[^ ^7^, ^42^, ^94^, ^96^, ^97^ ^]^ A total of 8% of the studies were conducted in more than one continent.^[^ ^38^, ^48^, ^55^, ^66^, ^71^, ^75^, ^76^, ^91^ ^]^ The region was not specified in 40% of the studies.^[^ ^10^, ^11^, ^22^, ^23^, ^24^, ^26^, ^29^, ^30^, ^31^, ^32^, ^33^, ^36^, ^37^, ^49^, ^51^, ^56^, ^57^, ^58^, ^59^, ^61^, ^62^, ^63^, ^68^, ^69^, ^70^, ^72^, ^73^, ^74^, ^81^, ^88^, ^90^, ^92^, ^93^, ^101^, ^104^, ^105^, ^106^, ^108^, ^109^ ^]^ The studies were carried out in various healthcare settings. A substantial proportion, 41%, of the studies were conducted in hospital settings.^[^ ^7^, ^19^, ^21^, ^24^, ^27^, ^31^, ^33^, ^34^, ^39^, ^40^, ^45^, ^46^, ^50^, ^54^, ^60^, ^65^, ^68^, ^72^, ^76^, ^77^, ^80^, ^82^, ^83^, ^84^, ^86^, ^87^, ^88^, ^95^, ^96^, ^97^, ^98^, ^99^, ^100^, ^102^, ^103^, ^107^, ^109^, ^110^, ^111^, ^112^ ^]^ Among these, seven studies were conducted in intensive care units (ICU), while a further seven took place in other specified hospital units.^[^ ^7^, ^21^, ^27^, ^31^, ^45^, ^72^, ^76^, ^77^, ^86^, ^96^, ^100^, ^103^, ^109^, ^112^ ^]^ Seven studies were carried out in hospital units that were not specified, while six studies took place across multiple units.^[^ ^19^, ^24^, ^39^, ^46^, ^60^, ^65^, ^82^, ^87^, ^95^, ^98^, ^107^, ^110^, ^111^ ^]^ Furthermore, six of the studies were conducted in surgical settings.^[^ ^50^, ^68^, ^80^, ^83^, ^84^, ^97^ ^]^ In contrast, 12 of the study sites were located in outpatient settings.^[^ ^38^, ^41^, ^43^, ^44^, ^47^, ^48^, ^64^, ^70^, ^71^, ^78^, ^91^, ^94^ ^]^ Three studies included both inpatients and outpatients.^[^ ^53^, ^55^, ^69^ ^]^ The setting of care was not specified in 43% of the studies.^[^ ^10^, ^11^, ^20^, ^22^, ^23^, ^25^, ^26^, ^28^, ^29^, ^30^, ^32^, ^35^, ^36^, ^37^, ^42^, ^49^, ^51^, ^52^, ^56^, ^57^, ^58^, ^59^, ^61^, ^62^, ^63^, ^66^, ^67^, ^73^, ^74^, ^75^, ^79^, ^81^, ^85^, ^90^, ^92^, ^93^, ^101^, ^104^, ^105^, ^106^, ^108^ ^]^

### 3.3 Identified drugs and drug classes associated with hypotensive effects

**Figure 2:**
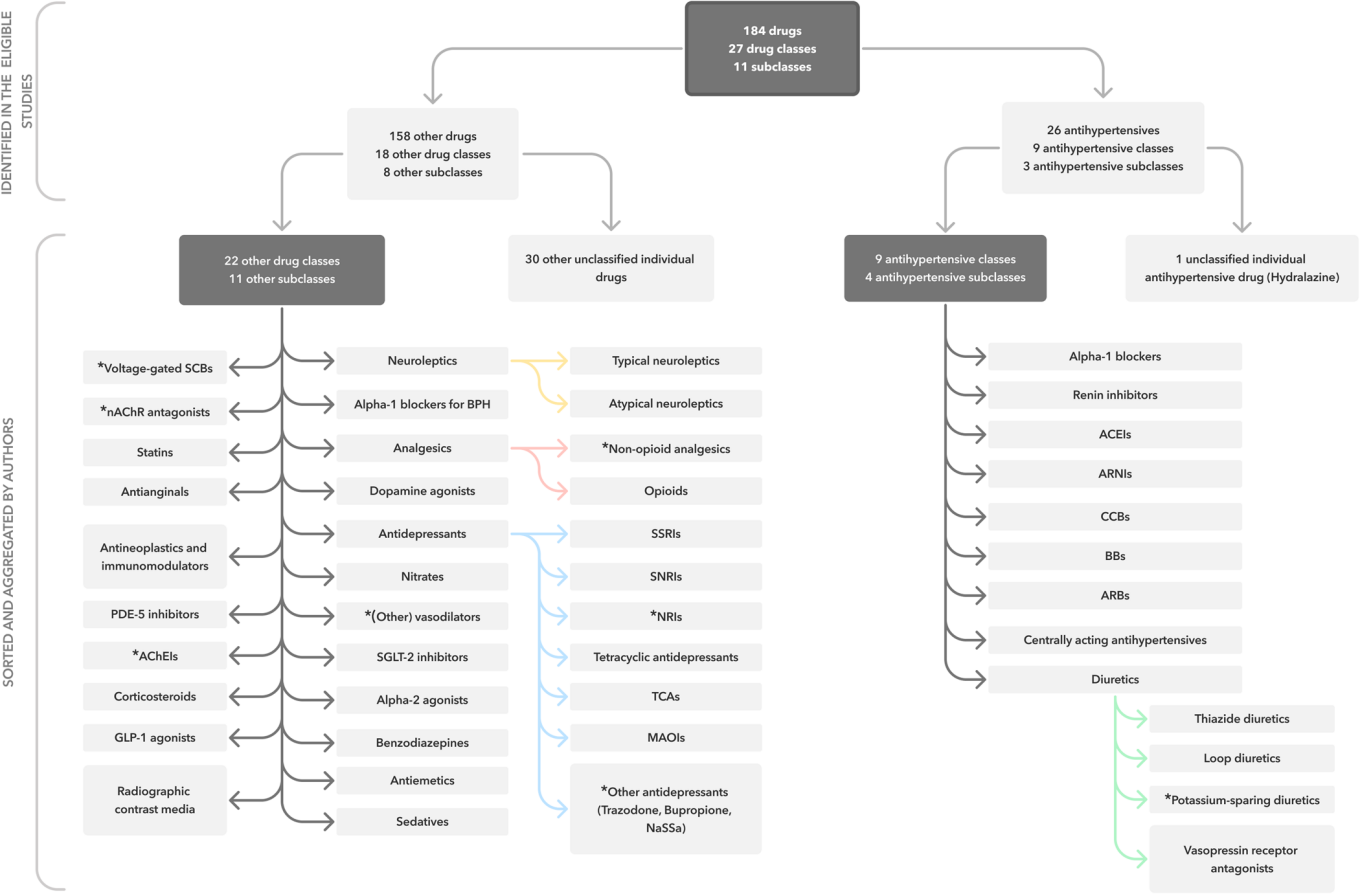
Identified drugs and drug classes. Drug classes added by the authors are marked with ‘*’. ACEIs, angiotensin‐converting enzyme inhibitors; AChEIs, acetylcholinesterase inhibitors; ARBs, angiotensin II receptor blockers; ARNIs, angiotensin receptor–neprilysin inhibitors; BBs, beta blockers; BPH, benign prostate hyperplasia; CCBs, calcium channel blockers; GLP‐1, glucagon‐like peptide 1; MAOIs, monoamine oxidase inhibitors; nAChR, nicotinic acetylcholine receptor; NaSSa, noradrenergic and specific serotonergic antidepressant; NRIs, norepinephrine reuptake inhibitors; PDE‐5, phosphodiesterase‐5; SCBs, sodium channel blockers; SGLT‐2, selective sodium glucose cotransporter‐2; SNRIs, selective norepinephrine reuptake inhibitors; SSRIs, selective serotonin reuptake inhibitors; TCAs, tricyclic antidepressant.

From the eligible studies, we extracted 184 individual drugs, 26 of which were antihypertensives. In addition to the individual drugs, nine antihypertensive classes and 18 other drug classes were identified. After the classification process by the reviewers, four additional other drug classes were formed as shown in Figure 2. All identified drugs and associated studies are listed in Supporting Information S1: Supplement S3.

#### 3.3.1 Antihypertensive agents

In total, 26 individual antihypertensive agents were identified and classified into nine antihypertensive classes, including four subclasses of diuretics (see Figure 2). Hydralazine was the only antihypertensive agent that could not be assigned to a drug class.^[^ ^76^, ^96^ ^]^

**Figure 3:**
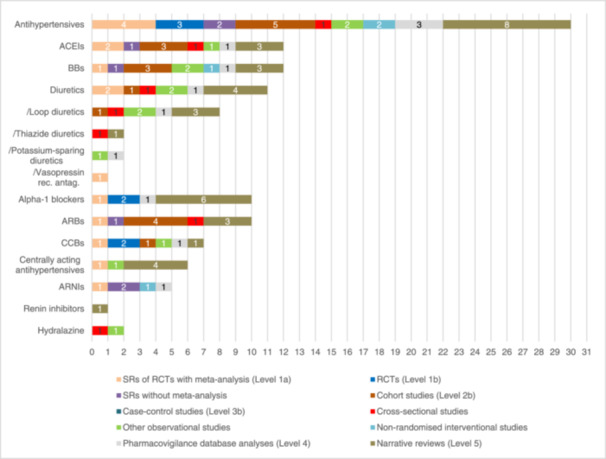
Number of studies per study type on antihypertensive drugs causing hypotension. Subclasses are marked with ‘/’. ACEIs, angiotensin‐converting enzyme inhibitors; ARBs, angiotensin II receptor blockers; ARNIs, angiotensin receptor–neprilysin inhibitors; BBs, beta blockers; CCBs, calcium channel blockers; RCTs, randomised controlled trials; rec. antag., receptor antagonist; SRs, systematic reviews.

Overall, 30% (30/100) of the eligible studies mentioned antihypertensive drugs in relation to hypotension.^[^ ^7^, ^10^, ^11^, ^17^, ^19^, ^20^, ^22^, ^26^, ^28^, ^33^, ^36^, ^41^, ^43^, ^49^, ^52^, ^54^, ^57^, ^61^, ^69^, ^70^, ^72^, ^73^, ^74^, ^76^, ^80^, ^81^, ^96^, ^99^, ^104^, ^106^ ^]^ The number of eligible studies for each antihypertensive class in conjunction with their study type is shown in Figure 3. Of the 30 studies on antihypertensive agents, 27% (8/30) were narrative reviews and 17% (5/30) were cohort studies.^[^ ^7^, ^11^, ^17^, ^19^, ^20^, ^43^, ^49^, ^57^, ^61^, ^72^, ^73^, ^99^, ^106^ ^]^ Among the reported antihypertensive classes, angiotensin‐converting enzyme inhibitors (ACEIs) (12/30), beta blockers (BBs) (12/30) and diuretics (11/30) were most frequently mentioned in the included publications.^[^ ^7^, ^10^, ^11^, ^17^, ^20^, ^22^, ^28^, ^36^, ^43^, ^49^, ^54^, ^70^, ^72^, ^73^, ^76^, ^96^, ^99^, ^104^ ^]^ Loop diuretics (*n* = 8) were the most commonly reported antihypertensive subclass.^[^ ^7^, ^11^, ^17^, ^28^, ^54^, ^73^, ^76^, ^96^ ^]^

All antihypertensive classes except renin inhibitors were analysed in one SR with MA, while ACEIs and diuretics were analysed in two SRs with MA.^[^ ^10^, ^22^, ^36^, ^81^ ^]^ Only alpha‐1 blockers and calcium channel blockers (CCBs, covering 1,4‐dihydropyridines [DHPs] and non‐DHPs) were represented in RCTs, making them the drug classes with the highest proportion of studies with a high level of evidence.^[^ ^41^, ^74^, ^80^ ^]^ BBs, ACEIs, diuretics, angiotensin II receptor blockers (ARBs), CCBs and centrally acting antihypertensives were represented in observational studies.^[^ ^7^, ^19^, ^20^, ^43^, ^54^, ^76^, ^96^, ^99^ ^]^ Renin inhibitors were the least reported drug class, with only one mention in a narrative review.^[^ ^72^ ^]^

When assessing the distribution of evidence levels, ACEIs, BBs, diuretics and CCBs were represented by at least six study types. Among the subclasses, whereas vasopressin receptor antagonists were analysed in an SR with MA, the other diuretic subclasses were only found in observational studies, database analyses and narrative reviews.^[^ ^7^, ^11^, ^17^, ^22^, ^28^, ^54^, ^73^, ^76^, ^96^ ^]^

In terms of study populations, many studies focused on older patients (either with a mean age of at least 60 years or solely including individuals of at least 50 years of age) or patients with comorbidity. A focus on older people was found in studies evaluating BBs (5/12), ACEIs (4/12), diuretics (4/11), ARBs (4/10), CCBs (2/7), centrally acting antihypertensives (1/6) and angiotensin receptor–neprilysin inhibitors (ARNIs) (1/5).^[^ ^7^, ^19^, ^20^, ^52^, ^54^, ^70^, ^74^, ^76^, ^96^, ^99^ ^]^ Notably, 3/5 ARNI studies included patients with heart failure, while one cohort study of ACEIs and ARBs focused on patients with acute kidney injury.^[^ ^33^, ^69^, ^99^, ^104^ ^]^

#### 3.3.2 Other drug classes

**Figure 4:**
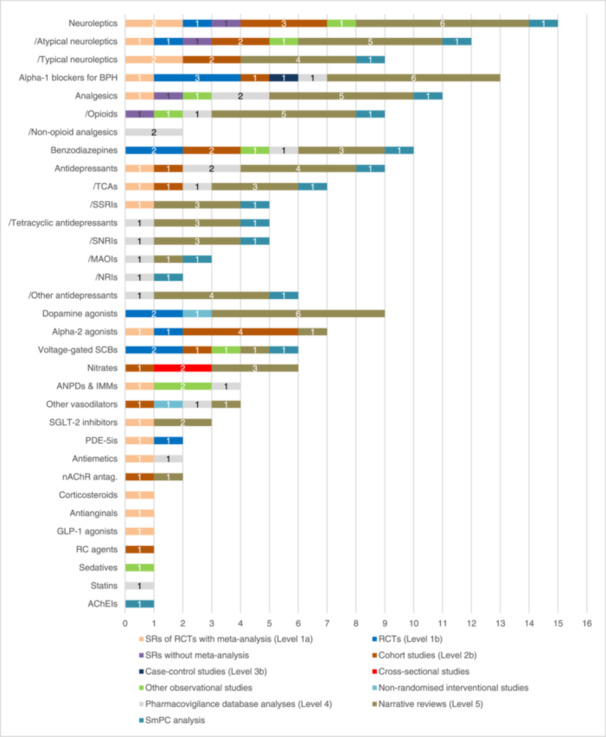
Number of studies per study type on other drug classes causing hypotension. Subclasses are marked with ‘/’. AChEIs, acetylcholinesterase inhibitors; ANPDs & IMMs, antineoplastic drugs & immunomodulators; antag., antagonist; BPH, benign prostate hyperplasia; GLP‐1, glucagon‐like peptide‐1; MAOIs, monoamine oxidase inhibitors; nAChR, nicotinic acetylcholine receptor; NRIs, noradrenaline reuptake inhibitors; other antidepressants: trazodone, bupropione, noradrenergic and specific serotonergic antidepressants; PDE‐5is, phosphodiesterase‐5 inhibitors; RC, radiographic contrast; RCTs, randomised controlled trials; SCBs, sodium channel blockers; SGLT‐2, selective sodium glucose cotransporter‐2; SNRIs, serotonine norepinephrine reuptake inhibitors; SRs, systematic reviews; SSRIs, selective serotonin reuptake inhibitors; TCAs, tricyclic antidepressants.

**Figure 5:**
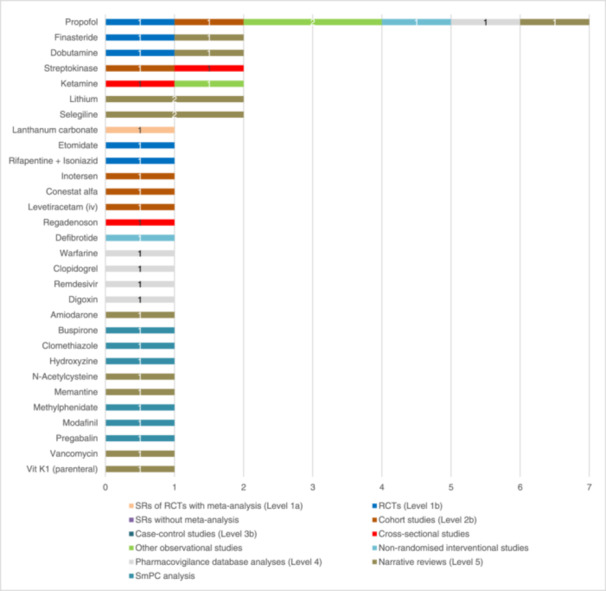
Number of studies per study type on other unclassified drugs causing hypotension iv, intravenous; RCTs, randomised controlled trials; SRs, systematic reviews; vit, vitamin.

Beyond the antihypertensive classes, we extracted 158 other individual drugs, 18 other drug classes and eight other subclasses. One hundred and twenty‐eight of these drugs were classified into 22 drug classes and 11 subclasses (see Figure 2). Figure 4 shows the number of eligible studies for each other drug class and the corresponding study types. Similarly, Figure 5 shows 30 individual drugs that were not classified. Overall, 12/22 other drug classes and 4/11 subclasses were represented in SRs with MA.^[^ ^10^, ^56^, ^88^ ^]^ Similarly, 13/22 drug classes and 4/11 subclasses were included in observational studies, while 12/22 drug classes and 9/11 subclasses were noted in narrative reviews.^[^ ^11^, ^17^, ^23^, ^27^, ^29^, ^30^, ^31^, ^32^, ^34^, ^35^, ^37^, ^40^, ^47^, ^49^, ^51^, ^57^, ^59^, ^61^, ^62^, ^63^, ^65^, ^68^, ^72^, ^76^, ^77^, ^78^, ^82^, ^84^, ^90^, ^92^, ^95^, ^96^, ^98^, ^100^, ^103^, ^106^, ^107^, ^108^, ^109^, ^112^ ^]^ The analysis of SmPCs of psychiatric medications conducted by Freudenmann et al. identified 6/22 other drug classes and 10/11 other subclasses.^[^ ^89^ ^]^

Of these, neuroleptics, alpha‐1 blockers for BPH, analgesics and antidepressants were mentioned in at least one SR with MA, while for benzodiazepines (BZDs) and dopamine agonists two RCTs provided the highest level of evidence.^[^ ^10^, ^21^, ^55^, ^83^, ^88^, ^91^ ^]^ Neuroleptics stood out with being mentioned in two SRs with MA.^[^ ^10^, ^88^ ^]^ Furthermore, alpha‐1 blockers and neuroleptics were also analysed in RCTs.^[^ ^45^, ^74^, ^85^, ^94^ ^]^ At the subclass level, selective serotonin reuptake inhibitors (SSRIs), TCAs and both typical and atypical neuroleptics were discussed in at least one SR with MA, while the other antidepressant subclasses were only mentioned in database analyses, narrative reviews or the SmPC analysis.^[^ ^10^, ^11^, ^32^, ^39^, ^49^, ^63^, ^88^, ^89^ ^]^ Nonopioid analgesics were only included in two database analyses.^[^ ^28^, ^67^ ^]^ Despite being the most commonly reported subclass, opioids (*n* = 9) were mostly included in lower levels of evidence, with the highest level of evidence being one SR without MA.^[^ ^101^ ^]^

In addition to these drug classes, several others were mentioned at least six times: alpha‐2 agonists (*n* = 7), voltage‐gated sodium channel blockers (SCBs) (*n *= 6) and nitrates (*n *= 6).^[^ ^10^, ^11^, ^17^, ^27^, ^40^, ^50^, ^60^, ^65^, ^72^, ^84^, ^89^, ^95^, ^96^, ^98^, ^109^, ^112^ ^]^ Alpha‐2 agonists and SCBs were reported by either RCTs or SRs with MA.^[^ ^10^, ^50^, ^60^ ^]^

The following drug classes were the least represented in the included studies and were only reported once: corticosteroids, antianginals, glucagon‐like peptide 1 (GLP‐1) agonists, radiographic contrast agents, sedatives, acetylcholinesterase inhibitors (AChEIs) and statins.^[^ ^10^, ^25^, ^56^, ^76^, ^89^, ^107^ ^]^ Antiemetics, nicotinic acetylcholine receptor (nAChR) antagonists and phosphodiesterase (PDE)‐5 inhibitors were mentioned twice in the included studies, while sodium‐glucose cotransporter (SGLT)‐2 inhibitors were mentioned in three publications.^[^ ^10^, ^28^, ^31^, ^62^, ^72^, ^79^, ^90^ ^]^ Vasodilators, antineoplastics and immunomodulators were reported in four studies.^[^ ^10^, ^23^, ^35^, ^44^, ^52^, ^58^, ^72^, ^100^ ^]^ Among the rarely mentioned drug classes, PDE‐5 inhibitors, GLP‐1 agonists, antineoplastics and immunomodulators, antiemetics, SGLT‐2 inhibitors, corticosteroids and antianginals were mentioned in relation to hypotension in one SR with MA.^[^ ^10^, ^56^ ^]^ Tadalafil, a PDE‐5 inhibitor, combined with tamsulosin was associated with hypotension in an RCT involving healthy volunteers.^[^ ^79^ ^]^ Among these rarely mentioned drug classes, AChEIs, statins, sedatives, radiographic contrast agents, nAChR antagonists and vasodilators were only mentioned in studies with a low level of evidence.^[^ ^25^, ^31^, ^35^, ^44^, ^52^, ^72^, ^76^, ^89^, ^107^ ^]^ Atypical and typical neuroleptics, opioids, BZDs, voltage‐gated SCBs, AChEIs and all identified subclasses of antidepressants were identified in the SmPC analysis by Freudenmann et al.^[^ ^89^ ^]^ Of the 22 drug classes, AChEIs were only mentioned in the SmPC analysis.^[^ ^89^ ^]^

#### 3.3.3 Details on frequently mentioned drug classes

A total of 9/22 drug classes were observed in at least six publications.^[^ ^10^, ^11^, ^17^, ^21^, ^26^, ^27^, ^28^, ^29^, ^30^, ^32^, ^34^, ^37^, ^39^, ^40^, ^45^, ^47^, ^49^, ^50^, ^51^, ^55^, ^57^, ^59^, ^60^, ^61^, ^63^, ^65^, ^66^, ^67^, ^68^, ^71^, ^72^, ^74^, ^76^, ^77^, ^78^, ^82^, ^83^, ^84^, ^85^, ^88^, ^89^, ^91^, ^92^, ^93^, ^94^, ^95^, ^96^, ^98^, ^101^, ^103^, ^106^, ^108^, ^109^, ^112^ ^]^ In the following section, special features of these drug classes are presented:

Among *neuroleptics*, atypical neuroleptics (*n *= 12) were mentioned more often than typical neuroleptics (*n *= 9), with five of the studies on atypical neuroleptics being narrative reviews and one being an SmPC analysis.^[^ ^11^, ^51^, ^59^, ^63^, ^72^, ^89^ ^]^ The atypical neuroleptics olanzapine, clozapine, quetiapine and risperidone were each mentioned in at least six publications, while risperidone and blonanserine were the subject of one RCT.^[^ ^11^, ^51^, ^59^, ^63^, ^65^, ^72^, ^77^, ^82^, ^85^, ^89^, ^93^ ^]^ Among the typical neuroleptics, chlorpromazine was mentioned most frequently (*n *= 5), while only prochlorperazine in one SR with MA had high‐level evidence.^[^ ^11^, ^51^, ^63^, ^72^, ^77^, ^88^ ^]^

*Alpha‐1 blockers* for BPH (*n* = 13) were frequently found in studies with a high level of evidence, including one SR with MA and three RCTs.^[^ ^10^, ^45^, ^74^, ^94^ ^]^

*Analgesics* were categorised as nonopioid analgesics (*n* = 2) and opioids (*n *= 9).^[^ ^10^, ^11^, ^17^, ^28^, ^29^, ^30^, ^67^, ^72^, ^76^, ^89^, ^101^ ^]^ Fentanyl (*n* = 3) and morphine (*n* = 4) were the most commonly reported analgesics.^[^ ^11^, ^28^, ^29^, ^30^, ^76^ ^]^ Tapentadol was mentioned in an SR without MA and had the highest evidence level within individual analgesics.^[^ ^101^ ^]^ The SmPC analysis identified buprenorphine, buprenorphine/naloxone, methadone, levomethadone and naltrexone in relation to hypotension.^[^ ^89^ ^]^ The nonopioid acetaminophen, also known as paracetamol, was identified in analyses of the FAERS and KAERS databases.^[^ ^28^, ^67^ ^]^

*BZDs* were reported with indications such as alcohol withdrawal syndrome, long‐term sedation, acute behavioural disorders and cardiac surgery.^[^ ^21^, ^83^, ^98^, ^103^ ^]^ A total of 60% (*n *= 6) of the BZD studies were conducted in the hospital setting.^[^ ^21^, ^68^, ^76^, ^83^, ^98^, ^103^ ^]^ Midazolam (*n* = 6) was the most commonly reported BZD, included in one RCT in the intensive care setting.^[^ ^21^ ^]^ Another RCT investigated remimazolam in cardiac surgery.^[^ ^83^ ^]^ Six out of the eight individual BZDs identified in our review (lorazepam, oxazepam, flurazepam, diazepam, bromazepam and nitrazepam) were reported in the SmPC analysis by Freudenmann et al.^[^ ^89^ ^]^

Of the studies representing *antidepressants* (*n* = 9), more than half (*n *= 5) were narrative reviews or the SmPC analysis.^[^ ^11^, ^32^, ^49^, ^63^, ^89^ ^]^ Antidepressants were categorised into the subclasses TCAs (*n* = 7), SSRIs (*n* = 5), serotonin norepinephrine reuptake inhibitors (SNRIs) (*n *= 5), tetracyclic antidepressants (*n *= 5), monoamine oxidase inhibitors (MAOIs) (*n* = 3) and norepinephrine reuptake inhibitors (NRIs) (*n* = 2). Notably, only one SR with MA analysed antidepressants, focusing on TCAs and SSRIs, representing their only mention in high‐level evidence.^[^ ^10^ ^]^ Venlafaxine was the individual antidepressants most frequently observed.^[^ ^11^, ^32^, ^39^, ^63^, ^89^ ^]^ According to an SmPC analysis, amitriptyline, trimipramine (TCAs) and tranylcypromine (MAOI) were the antidepressants most strongly associated with hypotensive effects (frequency of the ADE ‘very often’, >10%).^[^ ^89^ ^]^

Within *dopamine agonists*, which are widely used in Parkinson's disease (PD), amantadine was the most commonly reported drug, appearing in two RCTs and one narrative review.^[^ ^55^, ^91^, ^92^ ^]^

Six out of seven studies of *alpha‐2 receptor agonists* focused on dexmedetomidine.^[^ ^40^, ^50^, ^65^, ^72^, ^98^, ^109^ ^]^ Two of these cohort studies examined elderly patients, with one study including participants with a mean age of 55 years, while the other included those aged 75 years and older.^[^ ^65^, ^98^ ^]^ Three of the studies, specifically one RCT and two cohort studies, included patients with specific comorbidity such as sepsis, alcohol withdrawal syndrome and gynaecological cancer.^[^ ^50^, ^98^, ^109^ ^]^ Six studies, including four cohort studies, were conducted on inpatient populations.^[^ ^40^, ^50^, ^65^, ^72^, ^98^, ^109^ ^]^

Of the six studies of SCBs in connection with hypotension, which are widely used as anticonvulsants or local anaesthetics, two RCTs and two observational studies were carried out in hospital settings.^[^ ^50^, ^60^, ^84^, ^95^ ^]^

#### 3.3.4 Unclassified other drugs

Thirty drugs remained unclassified and are shown in Figure 5. Among these, propofol stood out, being mentioned in seven studies, including one RCT.^[^ ^21^, ^28^, ^72^, ^76^, ^97^, ^102^, ^109^ ^]^ Three of these studies were conducted in the ICU, one in the surgical context and one in the emergency department.^[^ ^21^, ^76^, ^97^, ^102^, ^109^ ^]^ Of the other drugs, finasteride, ketamine, streptokinase, dobutamine, selegiline and lithium were mentioned twice.^[^ ^11^, ^17^, ^42^, ^57^, ^63^, ^72^, ^74^, ^86^, ^87^, ^110^, ^112^ ^]^ All other unclassified drugs were only noted once in connection with hypotension. Of these, only lanthanum carbonate was associated with hypotension in one SR with MA focusing on patients with chronic kidney disease, while etomidate and rifapentine in combination with isoniazid were analysed in one RCT each.^[^ ^24^, ^38^, ^83^ ^]^ Clopidogrel, warfarin, propofol and digoxin were reported to cause hypotension in a FAERS analysis.^[^ ^28^ ^]^ Six of the 30 unclassified other drugs were only mentioned in the SmPC analysis by Freudenmann et al.^[^ ^89^ ^]^

#### 3.3.5 Drugs mentioned in relation to OH

Although our main focus was on drugs associated with general hypotension, 21% (*n *= 20) of the included literature described connections with OH.^[^ ^10^, ^11^, ^26^, ^32^, ^37^, ^49^, ^51^, ^55^, ^63^, ^67^, ^71^, ^74^, ^77^, ^79^, ^85^, ^88^, ^91^, ^92^, ^106^, ^108^ ^]^ Specifically, at least one source associated 7/9 antihypertensive classes and 14/22 other drug classes or their individual drugs with OH. Within antihypertensives, BBs, diuretics, ACEIs, ARBs, CCBs, alpha‐1 blockers and centrally acting antihypertensives were associated with OH.^[^ ^10^, ^11^, ^26^, ^49^, ^74^, ^106^ ^]^ All of the other drug classes with at least six mentions were also found to be associated with OH.^[^ ^10^, ^11^, ^26^, ^32^, ^37^, ^49^, ^51^, ^55^, ^63^, ^67^, ^71^, ^74^, ^77^, ^85^, ^88^, ^91^, ^92^, ^106^, ^108^ ^]^ Certain ones stood out in terms of the number of associations with OH. For example, seven out of nine studies of dopamine agonists reported a connection to OH.^[^ ^11^, ^37^, ^55^, ^71^, ^91^, ^92^, ^108^ ^]^ Similarly, more than half of the studies (5/9) on antidepressants associated this drug class or its individual drugs with OH.^[^ ^10^, ^11^, ^32^, ^49^, ^63^ ^]^ Moreover, almost half of the trials (7/15) of neuroleptics or their individual drugs reported an association with OH.^[^ ^10^, ^11^, ^51^, ^63^, ^77^, ^85^, ^88^ ^]^ The same applies to alpha‐1 blockers for BPH with five out of 13 studies on OH.^[^ ^10^, ^11^, ^26^, ^74^, ^106^ ^]^ The individual drugs or drug classes mentioned in connection with OH are labelled in Supporting Information S1: Supplement S3.

## 4 DISCUSSION

### 4.1 Summary of findings

This review shows that hypotension is a potential adverse effect not only of antihypertensive drugs but also of a wide range of other drugs, demonstrating the diverse nature of drug‐induced hypotension. Within antihypertensives, ACEIs, BBs and diuretics were the most frequently mentioned. Beyond antihypertensives, the most commonly reported drug classes were neuroleptics, alpha‐1 blockers, analgesics (mainly opioids), BZDs, antidepressants and dopamine agonists.

### 4.2 Mechanism of action and its relevance

The hypotensive effects of most of the drugs identified in our review can be explained by three main pharmacological mechanisms: reduced sympathetic activity, vasodilation and diuresis. Sympathetic inhibitors, such as alpha‐1 blockers, BBs, neuroleptics and TCAs, were frequently mentioned in our review. BBs reduce cardiac output by blocking beta‐adrenergic receptors, while alpha‐1 blockers, neuroleptics and tricyclic and tetracyclic antidepressants inhibit alpha‐1 receptors, thereby reducing sympathetic tone and vasoconstriction.^[^ ^10^, ^11^, ^63^, ^113^, ^114^ ^]^ A meta‐analysis by Bhanu et al. showed a six‐ to sevenfold increased odds of OH with BBs and TCAs compared with placebo, while alpha‐blockers, atypical neuroleptics and centrally acting antihypertensives were associated with up to a twofold increased odds of OH, highlighting the important impact of sympathetic inhibition on hypotension.^[^ ^10^ ^]^ On the contrary, vasodilating drug classes such as CCBs, ARBs, nitrates, SSRIs and dopamine agonists were less frequently mentioned in our review, suggesting that their hypotensive effects may be less pronounced.^[^ ^10^, ^115^, ^116^, ^117^ ^]^ This is supported by Bhanu et al.'s meta‐analysis showing that vasodilating drug classes did not show a significant difference in odds of OH compared with placebo.^[^ ^10^ ^]^ This aligns with a narrative review that reported that SSRIs cause OH less frequently than TCAs.^[^ ^11^ ^]^ Consistent with the existing literature on OH, our review highlights that drugs inhibiting sympathetic activity are often more prominent in hypotensive ADEs than those causing direct vasodilation.^[^ ^10^, ^118^ ^]^ This highlights the importance of understanding the underlying pharmacological mechanisms when assessing the hypotensive risks associated with different drug classes.

Moreover, an understanding of the mechanism of action enables healthcare professionals to anticipate potential hypotensive effects and implement preventive strategies, such as dose titration. Dose titration was addressed in several studies of this review, and is also recommended in the European Society of Cardiology (ESC) guideline for the management of hypertension.^[^ ^33^, ^41^, ^43^, ^49^, ^52^, ^69^, ^119^ ^]^ This approach allows a controlled initiation of these drugs, which significantly reduces the risk of hypotension. Also, based on the primary pharmacological mechanisms and SmPC information, healthcare providers may be more vigilant when administering drug classes known to cause hypotension, increasing the likelihood of identifying drug‐induced hypotension and gaps in awareness of drugs less well known to cause hypotension. This may contribute to the frequent mention of well‐known antihypertensive drugs such as antihypertensives, alpha‐1 blockers and antipsychotics. On the other hand, radiographic contrast agents, antineoplastics and GLP‐1 agonists were not frequently mentioned in our review, possibly because hypotension is not expected based on their primary mechanisms of action.^[^ ^120^, ^121^ ^]^ These aspects demonstrate how pre‐existing knowledge can influence the identification of ADEs. As a result, hypotension from drugs known to be hypotensive may be less clinically impactful if properly managed, while drugs that are less well known may warrant further attention.

### 4.3 Correlation with frequency of prescription

Some of the most commonly reported drugs in our review are among the most commonly prescribed drugs, such as antidepressants and certain antihypertensives, including ARBs, ACEIs, diuretics, CCBs and BBs.^[^ ^119^, ^122^, ^123^ ^]^ The frequent mention of these drugs and their occurrence in analyses of pharmacovigilance databases in our review suggests a possible correlation between their frequency of prescription and the reporting rates of hypotension, as drugs with a larger user population are more likely to have their ADEs reported.^[^ ^28^, ^39^, ^66^ ^]^ Therefore, the widespread use of these antihypertensives and antidepressants makes their frequent mention in our review expected and highlights the already increased awareness of hypotension as an ADE of these commonly used drugs.

On the other hand, we found that less commonly prescribed drugs, such as AChEIs, nAChR antagonists, PDE‐5 inhibitors and renin inhibitors, were reported less frequently in our review.^[^ ^123^ ^]^ This may reflect their limited use in clinical practice, resulting in fewer reports of hypotension as an ADE, highlighting the need for further investigation of less commonly prescribed drugs that may also pose a risk of hypotension.^[^ ^123^ ^]^ However, the less frequent mention of these drug classes might also indicate that there is already a high level of awareness of their hypotensive effects and that the mechanisms are so well understood that less research has been conducted in this area after 2012. An alternative explanation for the lower frequency of reports could be that they either cause hypotension less frequently or with a milder effect compared to more frequently mentioned drugs.

### 4.4 Confounding by indication and age

When evaluating ADEs, it is important to distinguish between drug‐induced events and symptoms of an underlying disease. In this review, this distinction is particularly relevant for dopamine agonists in the treatment of PD, a condition that inherently affects autonomic blood pressure regulation, leading to OH even in the absence of medication.^[^ ^124^, ^125^, ^126^ ^]^ While our review includes two RCTs suggesting an association between amantadine and hypotension, a meta‐analysis found no increased risk of OH with dopamine agonists compared with placebo.^[^ ^55^, ^91^, ^127^ ^]^ This discrepancy highlights the importance of considering confounding by PD when interpreting these results.

A predominance of older populations has been found in studies investigating the effects of antihypertensives and dexmedetomidine on hypotension.^[^ ^7^, ^19^, ^20^, ^52^, ^54^, ^65^, ^70^, ^74^, ^76^, ^96^, ^98^, ^99^ ^]^ A substantial number of these studies are observational in nature.^[^ ^7^, ^19^, ^20^, ^54^, ^65^, ^76^, ^96^, ^99^ ^]^ They lack randomisation and are therefore more susceptible to confounding, including age‐related factors.^[^ ^128^ ^]^ This is particularly important as older people are already predisposed to hypotension due to age‐related physiological changes, such as reduced cardiovascular resilience, as well as an increased likelihood of polymedication and comorbidity.^[^ ^129^, ^130^ ^]^ While the focus on older populations in these studies may reflect the primary user demographics for antihypertensives, it is important to note that these medications are also prescribed to younger individuals.^[^ ^131^, ^132^ ^]^ Similarly, dexmedetomidine is used for sedation in all age groups but has been reported to cause a higher incidence of hypotension in those over 65 years of age.^[^ ^133^, ^134^, ^135^ ^]^ Given the potential for confounding by age in these studies, the predominance of older age groups in these studies requires cautious interpretation, as this may limit the generalisability of these findings on antihypertensives and dexmedetomidine to younger age groups.^[^ ^136^, ^137^ ^]^

### 4.5 Influence of the healthcare setting

A substantial proportion of the included studies were conducted in hospitals, including ICU and surgical units. BZDs, voltage‐gated SCBs, dexmedetomidine and propofol were predominantly found in hospital‐based studies, reflecting their real‐world use as anaesthetics or sedatives, but leading to possible confounding.^[^ ^133^, ^138^, ^139^ ^]^ Inpatient populations often have highly variable comorbidity and are often exposed to polymedication, which may confound the observed association with hypotension.^[^ ^140^, ^141^ ^]^ In addition, the close monitoring and immediate visibility of ADEs in these settings may facilitate the observation of ADEs, possibly increasing the reporting rate compared with the long‐term use of drugs in the outpatient setting.^[^ ^142^, ^143^, ^144^ ^]^ In the surgical setting, hypotension can be caused by factors such as blood loss, patient positioning and vascular compression, independent of drugs.^[^ ^145^ ^]^ This is compounded by the known adverse event of hypotension following the induction of anaesthesia.^[^ ^146^ ^]^ BZDs are predominantly used in intensive care and surgical settings but also in the outpatient setting.^[^ ^21^, ^83^, ^147^ ^]^ This observation is consistent with an expert opinion, suggesting that the majority of evidence for BZDs comes primarily from intensive care settings, which may contribute to hypotension as discussed above.^[^ ^11^ ^]^ However, a cross‐sectional analysis showed that BZD users had a significantly lower baseline systolic blood pressure than nonusers, suggesting that the hypotensive effect of BZDs is independent of the healthcare setting.^[^ ^148^ ^]^

### 4.6 Potential literature gaps

Some drug classes, including AChEIs, statins, sedatives, radiographic contrast agents, nAChR antagonists, renin inhibitors and subclasses such as nonopioid analgesics, SNRIs, NRIs, MAOIs and tetracyclic antidepressants, have only been identified in low‐level evidence studies that lack randomisation and control groups, making the observed associations uncertain and not generalisable. The limited mention of certain drug classes or their association with hypotension at lower evidence levels could either indicate a lack of association or indicate evidence gaps in the current literature, highlighting the need for further research, ideally RCTs, to investigate the true relationship between these less commonly mentioned drugs and hypotension.

### 4.7 Strengths and limitations

The additive value of this ScR lies in its comprehensive and unrestricted literature search in different healthcare settings and study types, which not only broadens the understanding of drugs associated with hypotension but also complements and extends the findings of the SR on OH by Bhanu et al., making it the first comprehensive ScR in the international literature.^[^ ^10^ ^]^ Our ScR also provides additional evidence on drug‐induced OH compared with the SR by Bhanu et al., which exclusively incorporated RCTs.^[^ ^10^ ^]^ We identified three additional drug classes (BZDs, dopamine agonists, nitrates) and six subclasses (loop diuretics, thiazide diuretics, nonopioid analgesics, SNRIs, NRIs, tetracyclic antidepressants) in connection with OH that were not covered by Bhanu et al., highlighting the comprehensive scope of our review. Furthermore, our categorisation of the identified drugs according to their mechanism of action improves our understanding of their adverse effects. Although the nature of the ScR precludes a quantitative assessment of the evidence, we have compensated for this with an evidence map. However, some limitations should be noted. While our review provides a comprehensive qualitative mapping of the available evidence, we did not focus on the dose‐dependence of antihypertensive effects or the role of drug–drug interactions. These factors are of great importance in clinical practice, where underlying conditions, improper dose adjustments, and drug combinations have the potential to influence the likelihood and severity of hypotension. While this ScR was designed to identify the scope of drug‐induced hypotension in the context of standard therapeutic concepts, future research could explore hypotension as a consequence of medication errors in greater detail to enhance clinical relevance. Furthermore, our comprehensive inclusion of drugs that induced hypotension, even if the effect was less pronounced compared with alternative drugs, was intended to encompass a diverse range of evidence. However, it may be beneficial to differentiate between drugs that are more likely or more severe in causing hypotension. Although this was not the primary objective of our ScR, it provides a basis for future SRs to investigate this in a more targeted and quantitative manner. Despite a carefully developed search strategy, some relevant studies may have been missed if the term ‘adverse drug events’ was not mentioned. However, the absence of this term suggests that such studies may not be directly relevant to our primary research focus. In addition, our search period had to be restricted. Therefore, relevant studies outside this time frame may be missing. The publication period was limited to ensure the relevance of our review to current clinical practice and to focus on recent developments and therapies in this rapidly progressing field. Furthermore, we focused on studies that showed a positive association between drugs and hypotension. Studies in which drugs did not show this effect were not taken into account. Although potentially limiting the scope, this decision was intentional to maintain a focused approach to our research question of causative drugs.

## 5 CONCLUSIONS

This review provides a comprehensive overview of antihypertensive and nonantihypertensive drugs that induce hypotension, leading to an increased awareness and understanding of drug‐induced hypotension. Underrepresented drug classes, such as statins and sedatives, warrant further investigation. In addition, future research could focus on hypotension due to medication errors, such as inappropriate dosage or drug–drug interactions, and potentially confounding or biasing factors, such as PD or highly monitored hospital settings. Another gap could be filled by an SR focusing on general hypotension, including MAs to compare the risks of commonly used drugs.

## CONFLICTS OF INTEREST STATEMENT

The authors declare no conflicts of interest.

## Supporting information

Supporting information.

## Data Availability

All data supporting the findings of this study are available within the paper and its supplementary information.
