# Supplementary material for: Identification of potentially causative drugs associated with hypotension: A scoping review
Source: Arch Pharm (Weinheim). 2024 Nov 28;358(1):e2400564. doi: 10.1002/ardp.202400564 (PMC11704057; doi:10.1002/ardp.202400564)
Supplement: Supplementary file 1 — Supporting information. [file ARDP-358-e2400564-s001.pdf]

Journal: Archiv der Pharmazie

Identification of potentially causative drugs associated with hypotension: A scoping review

SUPPLEMENT S1: Scoping review protocol

Nurunnisa Sari [1,2], Ulrich Jaehde [3] and Anna Maria Wermund [3]*

[1] Institute for Medical Information Processing, Biometry and Epidemiology - IBE, LMU Munich, Germany

[2] Pettenkofer School of Public Health, Munich, Germany

[3] Department of Clinical Pharmacy, Institute of Pharmacy, University of Bonn, Germany

*Corresponding author. E-mail: a.wermund@uni-bonn.de

Table of contents

1 Background 2
2 Objectives and review question 2
3 Methods 3
3.1 Eligibility criteria 4
3.2 Intended information sources 5
3.3 Search Strategy 5
3.4 Study/source of evidence selection 6
3.5 Data extraction 7
3.6 Data analysis and presentation 8
4 References 9

1 Background

Why is this research necessary?

- Hypotension can be harmful to the patient as it is a cause of morbidity and mortality. Complications of untreated hypotension are dangerous and can result in death (1).
- Study of hospitalisation data (Australia, England, Wales) shows significant increase of hypotension-related admission rates between 1999-2020 (168%, 398%, 149%) (2).
- A considerable proportion of hypotension-related hospital admissions are drug-related and hypotension is a common preventable adverse drug event (ADE) (2).
- Knowledge of potentially causative drugs for hypotension can lead to more tailored and proactive management strategies (e.g. risk models, clinical decision support systems).
- Many drugs have been identified as potential causative drugs, but a comprehensive overview of the evidence base is still lacking, as many reviews have focused on orthostatic hypotension (OH) or were narrative. A systematic review (SR) from 2021 focused only on OH and limited its search to randomized controlled trials (RCTs) (3).

Why is a scoping review (ScR) the appropriate method?

- To report on the types of evidence & construct an evidence map, leading to a summary of the existing evidence regardless of quality
- To identify evidence gaps
- To supplement the SR from 2021 on OH (3).

2 Objectives and review question

To provide a broad overview of drugs causing hypotension, without focusing on specific conditions, care settings or types of studies, this scoping review aims to identify potential causative drugs for hypotension in adults and to construct a comprehensive evidence map.

Research question: *What drugs have been associated with hypotension in adults and what type of evidence supports these associations?*

3 Methods

The review will be conducted in accordance with the JBI methodology for scoping reviews and the Preferred Reporting Items for Systematic Reviews and Meta-Analyses extension for Scoping Reviews (PRISMA-ScR) (4, 5).

Previous researches on ADEs and hypotension will be identified to collect suitable keywords/phrases.

Inclusion and exclusion criteria will be adapted after an initial search. Modifications of the search strategy/eligibility criteria during the review process are possible (if necessary).

3.1 Eligibility criteria

Table S1 Inclusion and exclusion criteria

	Inclusion criteria	Exclusion criteria
**Population**	Age ≥ 18 years	• P1: < 18 years<br>• P2: Pregnancy
**Concept = Event**	• Hypotension as a side effect	• E1: Hypotension as a symptom of another ADE (e.g. anaphylaxis, capillary leak syndrome, hypersensitivity, infusion-related reactions)<br>• E2: Drug-drug interactions or combination therapies (except for fixed-dose drug combinations, including e.g. tadalafil + tamsulosin, buprenorphine + naloxone, sacubitril + valsartan)<br>• E3: Drug toxicity due to overdose, overuse, poisoning, medication errors, drug abuse<br>• E4: Medical devices, excipients<br>• E5: Herbal medicinal products, traditional Chinese medicine<br>• E6: Hypotension not as ADE (e.g., drug treatment of hypotension)<br>• E7: No difference from placebo
**Context**	• Any care setting	• C0: Poison centres
**Study**	• Phase II/III – IV clinical trials<br>• All types of reviews<br>• RCTs<br>• Non-comparative/non-controlled experimental studies (single-arm studies)<br>• Observational studies (case-control, cross-sectional, cohort studies); prospective and retrospective designs<br>• Retrospective analysis of pharmacovigilance database data	• S1: No original results for drugs that cause hypotension, except for narrative reviews<br>• S2: Preclinical studies; animal studies; phase I-II clinical trials<br>• S3: Case reports and case series (except for retrospective analyses of pharmacovigilance databases)<br>• S4: No association of hypotension with a certain drug<br>• S5: Only registration in clinical trial databases (e.g. clinicaltrials.gov) without results<br>• S6: Language other than English or German<br>• S7: Conference abstracts

3.2 Intended information sources

Table S2 Information sources

Data Source	Inclusion	Reason for exclusion
**Medline via Pubmed**	Yes	
**Embase via OVID**	Yes	
**Cochrane Library**	Yes	
**Google Scholar**	No	Structured search is not possible
**SIDER**	No	Abundance of unverified data<br>(660 drugs with side effect hypotension)
**Web of Science**	No	Lack of time
**Scopus**	No	Lack of time
**Tertiary literature (Anne Lee about ADR)**	Yes	

- Cochrane standard: MEDLINE (PubMed), EMBASE (via Ovid) and Cochrane Library (6)
- Languages: English, German
- Time limitation: 1 January 2013 to 25 May 2023 (10 years)
- Detection of duplicates by “Rayyan” (7)

3.3 Search Strategy

As an initial search, the first 100 results in PubMed were screened in order to adjust the inclusion and exclusion criteria. The following exclusion criteria were added after the initial search:

- C1: Hypotension as a symptom of another ADE (e.g. anaphylaxis, capillary leak syndrome, hypersensitivity, infusion-related reactions)
- S5: Only registration in clinical trial databases (e.g. clinicaltrials.gov) without results
- S4: No association of hypotension with a certain drug
- C7: No difference from placebo

The search strategy was developed for MEDLINE and adapted accordingly for EMBASE and the Cochrane Library. Text word from Pubmed was translated into ti,ab,kw for Embase.

The final search strategy after the initial search (25 May 2023) is presented in Table 3.

Table S3 Final search terms

Concept	Subject Headings	Text Word Search
**Hypotension**	Hypotension	hypotens*
**ADE**	MeSH:<br>drug related side effects and adverse reactions<br><br>Emtree:<br>adverse drug reaction OR side effect	drug related side effect* OR<br>adverse drug event* OR<br>adverse drug reaction* OR<br>adverse drug effect* OR<br>drug-induced OR<br>drug-related OR<br>drug side effect* OR<br>drug adverse reaction* OR<br>drug event*<br><br>*In Pubmed as Text Word*<br>*In OVID as ti,ab,kw*
**Exclusion**	plants, medicinal OR<br>plant extract OR<br>phytotherapy OR<br>pregnancy OR<br>herbal medicine OR<br>anaphylaxis OR<br>hypersensitivity OR<br>animals<br><br>Only in OVID: "allergy"<br><br>*in Pubmed as Major topic*<br>*in OVID as Focus*	herbal medicine* OR<br>plant extract* OR<br>phytotherap* OR<br>gestation* OR<br>pregnan* OR<br>allerg* OR<br>anaphylaxis OR<br>anaphylact*<br>hypersensitivit*<br>child*<br>infant*<br>pediatric*<br>animal*<br>rat<br><br>*In Pubmed & OVID as Title*

3.4 Study/source of evidence selection

Initially, two independent reviewers (AMW and NS) will screen the literature for relevance of titles/abstracts. In case of disagreement, a third reviewer (UJ) will decide on relevance. If a publication is considered potentially relevant, it will proceed to the full-text screening stage. AMW and NS will conduct the full-text screening of the literature identified as potentially relevant. Again, in case of disagreement, a third reviewer (UJ) will decide on relevance. The screening process will be done using Rayyan (7).

The PRISMA flowchart will be used to summarise the selection of evidence, which also contains reasons for exclusions during full text screening (4).

3.5 Data extraction

NS will extract the relevant information from the included publications, AMW will check a sample of this information. The following data items will be extracted into data extraction sheets using Microsoft Excel™ (Version 2019, Microsoft Corporation, Redmond, USA):

- First author
- Title
- Year of publication
- Type of evidence/publication (levels of evidence see Table 4)
- Healthcare setting
- Country
- Population (age and special conditions)
- Drugs and drug classes associated with hypotension

Types of settings:

- Hospital/Inpatient:
    - Admission/Emergency department
    - Discharge
    - During stay (General ward)
    - During stay (ICU)
    - During stay (surgery)
    - During stay (other ward)
    - During stay (multiple units)
- Ambulatory/Outpatient
- Inpatient + outpatient
- No information provided

Table S4 Levels of evidence, graded/listed by decreasing strength of evidence

Level of evidence according to the Oxford Centre for EbM	***Study type***
**1a**	Systematic reviews of randomised controlled studies with meta-analysis
**1b**	Randomised controlled studies
**[a]**	Systematic reviews without meta-analysis
**2b**	Cohort studies
**3b**	Case-control studies
**[a]**	Cross-sectional studies
**[a]**	Other observational studies
**[a]**	Non-randomised interventional studies
**4**	Case series (in our case pharmacovigilance database analyses)
**5**	Expert opinions (in our case mainly narrative reviews)

[a] no representation in Oxford Centre for EbM

"High level of evidence" = systematic reviews of RCTs with meta-analysis or RCTs themselves.

3.6 Data analysis and presentation

The extracted drugs will be grouped into superordinate drug classes according to their mechanism of action. These superordinate drug classes are either named in the literature or defined by the researchers based on the mechanism of action. Evidence levels will be assigned to the extracted drugs according to the evidence levels of the studies that mention them. All drugs that cannot be assigned to a drug class will be grouped under the term 'unclassified other drugs'. In addition, we will mark whether the extracted drugs or drug classes were reported to be associated with OH and whether they were part of the publication by Bhanu et al. (3). Stacked bar graphs will be used to visualise the number and distribution of publications across different levels of evidence for each drug class or drug (three different graphs: (1) antihypertensives, (2) other drug classes, (3) other unclassified drugs).

Mentions of individual drugs belonging to subclasses or drug classes are assigned to their respective classes.

4 References

(1) Sharma S, Hashmi MF, Bhattacharya PT. Hypotension. [Updated 2023 Feb 19]. In: StatPearls [Internet]. Treasure Island (FL): StatPearls Publishing; 2024. Available from: https://www.ncbi.nlm.nih.gov/books/NBK499961/

(2) Hemmo SI, Naser AY, Taybeh EO. Hospital Admission Due to Hypotension in Australia and in England and Wales. Healthcare (Basel). 2023;11(9):1210. doi: 10.3390/healthcare11091210.

(3) Bhanu C, Nimmons D, Petersen I, Orlu M, Davis D, Hussain H, Magammanage S, Walters K. Drug-induced orthostatic hypotension: A systematic review and meta-analysis of randomised controlled trials. PLoS Med. 2021;18(11):e1003821. doi: 10.1371/journal.pmed.1003821.

(4) Tricco AC, Lillie E, Zarin W, O'Brien KK, Colquhoun H, Levac D, Moher D, Peters MDJ, Horsley T, Weeks L, Hempel S, Akl EA, Chang C, McGowan J, Stewart L, Hartling L, Aldcroft A, Wilson MG, Garritty C, Lewin S, Godfrey CM, Macdonald MT, Langlois EV, Soares-Weiser K, Moriarty J, Clifford T, Tunçalp Ö, Straus SE. PRISMA Extension for Scoping Reviews (PRISMA-ScR): Checklist and Explanation. Ann Intern Med. 2018;169(7):467-473. doi: 10.7326/M18-0850.

(5) Peters MDJ, Godfrey C, McInerney P, Munn Z, Tricco AC, Khalil, H. Scoping Reviews (2020). Aromataris E, Lockwood C, Porritt K, Pilla B, Jordan Z, editors. JBI Manual for Evidence Synthesis. JBI; 2024. Available from: https://synthesismanual.jbi.global. https://doi.org/10.46658/JBIMES-24-09

(6) Higgins JPT, Thomas J, Chandler J, Cumpston M, Li T, Page MJ, Welch VA (editors). Cochrane Handbook for Systematic Reviews of Interventions version 6.5 (updated August 2024). Cochrane, 2024. Available from www.training.cochrane.org/handbook.

(7) Rayyan – Intelligent Systematic Review, Rayyan. Available online at: https://www.rayyan.ai/

Journal: Archiv der Pharmazie

Identification of potentially causative drugs associated with hypotension: A scoping review

SUPPLEMENT S2: Search strategy

Nurunnisa Sari [1,2], Ulrich Jaehde [3] and Anna Maria Wermund [3]*

[1] Institute for Medical Information Processing, Biometry and Epidemiology - IBE, LMU Munich, Germany

[2] Pettenkofer School of Public Health, Munich, Germany

[3] Department of Clinical Pharmacy, Institute of Pharmacy, University of Bonn, Germany

*Corresponding author. E-mail: a.wermund@uni-bonn.de

Table S1 *Search strategy*

Database	*Search strategy*
MEDLINE<br><br>(25 May 2023)	1. "drug related side effect*"[Text Word]<br>2. "adverse drug event*"[Text Word]<br>3. "adverse drug reaction*"[Text Word]<br>4. "adverse drug effect*"[Text Word]<br>5. "drug-induced"[Text Word]<br>6. "drug-related"[Text Word]<br>7. "drug side effect*"[Text Word]<br>8. "drug adverse reaction*"[Text Word]<br>9. "drug event*"[Text Word]<br>10. 1 OR 2 OR 3 OR 4 OR 5 OR 6 OR 7 OR 8 OR 9<br>11. "hypotens*"[Text Word]<br>12. 10 AND 11<br>13. "herbal medicine*"[Title]<br>14. "plant extract*"[Title]<br>15. “phytotherap*"[Title]<br>16. "gestation*"[Title]<br>17. “pregnan*” [Title]<br>18. "allerg*"[Title]<br>19. "Anaphylaxis"[Title]<br>20. "anaphylact*"[Title]<br>21. "hypersensitivit*"[Title]<br>22. "child*"[Title]<br>23. "pediatric*"[Title]<br>24. "infant*"[Title]<br>25. "animal*"[Title]<br>26. "rat"[Title]<br>27. 13 OR 14 OR 15 OR 16 OR 17 OR 18 OR 19 OR 20 OR 21 OR 22 OR 23 OR 24 OR 25 OR 26<br>28. 12 NOT 27<br>29. "drug related side effects and adverse reactions"[MeSH Terms]<br>30. "hypotension"[MeSH Terms]<br>31. 29 AND 39<br>32. "plants, medicinal"[MeSH Major Topic]<br>33. "plant extracts"[MeSH Major Topic]<br>34. "phytotherapy"[MeSH Major Topic]<br>35. "pregnancy"[MeSH Major Topic]<br>36. "Anaphylaxis"[MeSH Major Topic]<br>37. "hypersensitivity"[MeSH Major Topic]<br>38. "animals"[MeSH Major Topic]<br>39. 32 OR 33 OR 34 OR 35 OR 36 OR 37 OR 38<br>40. 31 NOT 39<br>41. 40 OR 28
EMBASE<br><br>(25 May 2023)	1. *side effect/ or *adverse drug reaction/<br>2. *hypotension/<br>3. 2 and 3<br>4. *pregnancy/ or *phytotherapy/ or *plant extract/ or *herbal medicine/ or *hypersensitivity/ or *allergy/ or *anaphylaxis/ or *animals/<br>5. 3 not 4<br>6. ("drug related side effect*" or "adverse drug event*" or "adverse drug reaction*" or "adverse drug effect*" or drug-induced or drug-related or

Database	*Search strategy*
	"Drug side effect*" or "drug adverse reaction*" or "Drug event*").ab,kw,ti.<br><br>7. (herbal medicine* or plant extract* or phytotherap* or gestation* or pregnan* or allerg* or anaphylaxis or anaphylact* or hypersensitivit* or animal* or rat or pediatric* or infant* or child*).ti.<br>8. hypotens*.ab,kw,ti.<br>9. 6 and 8<br>10. 9 not 7<br>11. 5 or 10
Cochrane Library<br>(4 July 2023)	1. (hypotens*):ti,ab<br>2. ("drug related side effects" OR "adverse drug events" OR "adverse drug reactions" OR "adverse drug effects" OR "drug-induced" OR "drug-related" OR “Drug side effects” OR “drug adverse reactions” OR “Drug events” OR "drug related side effect" OR "adverse drug event" OR "adverse drug reaction" OR "adverse drug effect" OR “Drug side effect” OR “drug adverse reaction” OR “Drug event”):ti,ab<br>3. 1 AND 2<br>4. ("herbal medicine" OR "plant extract" OR "plant extracts" OR "phytotherapy" OR "phytotherapeutic" OR "gestational" OR "gestation" OR "pregnant" OR "pregnancy" OR "allergy" OR "allergic" OR "Anaphylaxis" OR "anaphylactoid" OR "anaphylactic" OR “Hypersensitivity" OR “Children" OR "infant" OR "infants" OR "Pediatric" OR "Animal" OR "animals" OR "Rat"):ti<br>5. 3 NOT 4<br>6. MeSH descriptor: [Drug-Related Side Effects and Adverse Reactions] explode all trees<br>7. MeSH descriptor: [Hypotension] explode all trees<br>8. 6 AND 7<br>9. MeSH descriptor: [Pregnancy] this term only<br>10. MeSH descriptor: [Phytotherapy] this term only<br>11. MeSH descriptor: [Plant Extracts] this term only<br>12. MeSH descriptor: [Herbal Medicine] this term only<br>13. MeSH descriptor: [Anaphylaxis] this term only<br>14. MeSH descriptor: [Hypersensitivity] this term only<br>15. 9 OR 10 OR 11 OR 12 OR 13 OR 14<br>16. 9 NOT 15<br>17. 16 OR 5

Journal: Archiv der Pharmazie

Identification of potentially causative drugs associated with hypotension: A scoping review

SUPPLEMENT S3: Extracted drugs and drug classes

Nurunnisa Sari [1,2], Ulrich Jaehde [3] and Anna Maria Wermund [3]*

[1] Institute for Medical Information Processing, Biometry and Epidemiology - IBE, LMU Munich, Germany

[2] Pettenkofer School of Public Health, Munich, Germany

[3] Department of Clinical Pharmacy, Institute of Pharmacy, University of Bonn, Germany

*Corresponding author. E-mail: a.wermund@uni-bonn.de

Table S1 *Reported drugs (Drug classes added by the authors are marked with "*"; Abbreviations: AChE = acetylcholinesterase; BPH = benign prostate hyperplasia; CS = cohort studies, CCS = case-control studies; CSS = cross-sectional studies; GLP-1 = glucagon-like peptide-1; i = inhibitor; MA = meta-analysis; nAChR = nicotinic acetylcholine receptor; NRIS = non-randomised interventional studies; OBS = other observational studies; OH = orthostatic hypotension; PDE-5 = phosphodiesterase-5; PVG-DB = pharmacovigilance database analyses; RCTs = randomised controlled trials; SGLT-2 = sodium glucose linked transporter 2; SmPC = Summaries of Product Characteristic; SRs = systematic reviews)*

												Associated with OH[1]:	
Drug classes	***Drugs***	***SRs with MA of RCTs***	***RCTs***	***SRs without MA***	***CS***	***CCS***	***CSS***	***OBS***	***NRIS***	***PVG-DB***	***Narrative reviews/ SmPC analysis***	***Bhanu et al. [10]***	***Other study***
							ANTIHYPERTENSIVES						
**ANGIOTENSIN CONVERTING ENZYME INHIBITORS**		36, 10			99, 43						11, 17, 72	X	X
	Captopril			104							11		X
	Enalapril				7		96				11		X
	Lisinopril							76		28			
	Ramipril				99					28			
	Perindopril										11		X
**BETA BLOCKERS**		10		104	20, 43			54			11, 72	X	X
	Carvedilol			104	7					28	11		X
	Atenolol				7					28			
	Metoprolol			104				76		28			
	Nebivolol			104					70				
	Propranolol										49		X
	Labetalol							76					
**DIURETICS**		10									11, 17, 72	X/	X
LOOP DIURETICS								54			11, 17		X
	Furosemide				7		96	76		28	73		
THIAZIDE DIURETICS											11		X
	Hydrochloro-thiazide						96						
**POTASSIUM-SPARING DIURETICS*													
	Spironolactone							54		28			
	Eplerenone							54					
VASOPRESSIN RECEPTOR ANTAGONISTS		22											
**ALPHA-1 BLOCKERS**		10	74								11, 17, 57, 61, 72, 106	X	X
	Doxazosin		74							26	11, 57		X
	Prazosin		80								11, 106		X
**ANGIOTENSIN-II RECEPTOR BLOCKERS**		10		104	43, 99						11, 17, 72	X	X

Drug classes	Drugs	SRs with MA of RCTs	RCTs	SRs without MA	CS	CCS	CSS	OBS	NRIS	PVG-DB	Narrative reviews/ SmPC analysis	Associated with OH[1]: Bhanu et al. [10]	Other study
	Fimasartan				19								
	Losartan			104	7		96						
	Valsartan				7								
**CALCIUM CHANNEL BLOCKERS**		10									11	X	X
	Amlodipine		80		7					28			
	Nifedipine		41										
	Diltiazem							76					
**CENTRALLY ACTING ANTI-HYPERTENSIVES**		10									17	X	
	Clonidine							76			11, 49, 72		X
**ANGIOTENSIN RECEPTOR - NEPRILYSIN INHIBITORS**				104									
	Sacubitril + Valsartan	81		104, 33					69	52			
**RENIN INHIBITORS**											72		
**UNCLASSIFIED ANTI-HYPERTENSIVES**													
	Hydralazine						96	76					

OTHER DRUGS

Drug classes	Drugs	SRs with MA of RCTs	RCTs	SRs without MA	CS	CCS	CSS	OBS	NRIS	PVG-DB	Narrative reviews/ SmPC analysis	Associated with OH[1]: Bhanu et al. [10]	Other study
**NEUROLEPTICS**					77						11, 51, 72, 17		X
ATYPICAL NEUROLEPTICS		10									63	X	X
	Quetiapine			93	77						11, 51, 63, 72, 89		X
	Risperidone		85		77						51, 63, 72, 89		X
	Asenapine										63, 89		X
	Paliperidone										63, 89		X
	Iloperidone										63		X
	Lurasidone										63		X
	Sertindole										63, 89		X
	Zotepine										63		X
	Clozapine				77						11, 51, 59, 63, 72, 89		X
	Olanzapine				65, 77			82			11, 51, 63, 89		X
	Ziprasidone										51, 63, 89		X
	Amisulpride										89		
	Sulpiride										89		
	Aripiprazole										63, 89		X
	Blonanserin		85										

Drug classes	Drugs	SRs with MA of RCTs	RCTs	SRs without MA	CS	CCS	CSS	OBS	NRIS	PVG-DB	Narrative reviews/ SmPC analysis	Associated with OH[1]:	
												Bhanu et al. [10]	Other study
TYPICAL NEUROLEPTICS		10									63	X/	X
	Benperidol										89		
	Chlorpromazine				77						11, 51, 63, 72		X
	Haloperidol										11, 51, 89		X
	Droperidol				103						63		X
	Thioridazine										51, 63, 89		X
	Prochlorper-azine	88									63		X
	Levomepro-mazine										89		
	Perazine										89		
	Fluphenazine										63, 89		X
	Perphenazine										63		X
	Thiothixene										63		X
	Trifluoroperazine										63		X
	Pimozide										63		X
	Molindone										63		X
	Fluspirilene (i.m.)										89		
	Zuclopenthixol										89		
	Flupentixol										89		
	Chlorprothixene										89		
	Pipamperone										89		
	Loxapine										63, 89		X
**ALPHA-1 BLOCKERS FOR BPH**		10	74								11, 17, 57, 61, 72,106	X	X
	Alfuzosin		94			34				26	11, 106		X
	Terazosin					34				26	11, 57, 106		X
	Silodosin				47								
	Tamsulosin		45							26	11, 57, 106		X
**ANALGESICS**		10						76				X/	X
OPIOIDS											11, 17, 30, 72		X
	Buprenorphine										30, 89		
	Buprenorphine+ Naloxone										89		
	Fentanyl							76			29, 30		
	Morphine									28	11, 29, 30		X
	Naltrexone										89		
	Hydromorphone										11, 30		
	Methadone										30, 89		
	Levomethadone										89		
	Oxycodone										30		

Drug classes	Drugs	SRs with MA of RCTs	RCTs	SRs without MA	CS	CCS	CSS	OBS	NRIS	PVG-DB	Narrative reviews/ SmPC analysis	Associated with OH[1]: Bhanu et al. [10]	Other study
	Tramadol										30		
	Tapentadol			101									
	Hydrocodone										11		X
	Meperidine										11		X
** NON-OPIOID ANALGESICS*													
	Acetaminophen (Paracetamol)									28, 67			X
	Propacetamol									67			X
	Acetylsalicylic acid									28			
**BENZODIAZEPINES**											11, 17		X
	Midazolam		21		98, 103			76		28	68		
	Nitrazepam										89		
	Bromazepam										89		
	Diazepam										89		
	Flurazepam										89		
	Oxazepam										89		
	Remimazolam		83								68		
	Lorazepam				98			76		28	89		
**ANTIDEPRESSANTS**										39, 66	11, 63		X
TRICYCLIC ANTIDEPRESSANTS		10								39	11, 32, 63	X	X
	Clomipramine									39	32, 63		X
	Imipramine										63, 89		X
	Doxepin									39	32, 63, 89		X
	Opipramol										89		
	Trimipramine										63, 89		X
	Amitriptyline									39	32, 63, 89		X
	Amoxapine										63		X
	Desipramine										63		X
	Dosulepin										32		X
	Nortriptyline				78						32, 63, 89		X
SELECTIVE SEROTONIN REUPTAKE INHIBITORS (SSRIS)		10									11, 32, 63	X	X
	Citalopram										11, 63, 89		X
	Escitalopram										63		
	Fluoxetine										11, 32, 63, 89		X
	Paroxetine										11, 32, 63, 89		X
	Sertraline										11, 63, 89		X

												Associated with OH[1]:	
Drug classes	***Drugs***	***SRs with MA of RCTs***	***RCTs***	***SRs without MA***	***CS***	***CCS***	***CSS***	***OBS***	***NRIS***	***PVG-DB***	***Narrative reviews/ SmPC analysis***	***Bhanu et al. [10]***	***Other study***
	Fluvoxamine										32, 63, 89		X
	Vortioxetine										63		X
TETRACYCLIC ANTI-DEPRESSANTS											32, 63		X
	Mirtazapine									39	32, 63, 89		X
	Mianserin										32, 49, 89		X
	Maprotiline										32, 63, 89		X
	Protriptyline										63		X
SEROTONIN NOR-EPINEPHRINE REUPTAKE INHIBITORS (SNRIS)										39	11, 32, 63		X
	Duloxetine										11, 32, 63, 89		X
	Venlafaxine									39	11, 32, 63, 89		X
	Desvenlafaxine										63		X
	Milnacipran										89		
	Levomilnacipran										63		X
MONOAMINE OXIDASE INHIBITORS										39			
	Tranylcypromine										63, 89		X
	Moclobemide										89		
	Phenelzine										63		X
	Isocarboxazid										63		X
** NOR-EPINEPHRINE REUPTAKE INHIBITORS (NRIS)*													X
	Reboxetine									39	89		
	Atomoxetine										89		
** OTHER ANTI-DEPRESSANTS*													
	Trazodone										11, 32, 49, 89		X
	Bupropione										32, 63		X
	Noradrenergic and Specific Serotonergic Antidepressant (NaSSa)									39			
**DOPAMINE AGONISTS**											17, 72		
	Levodopa										11, 37		X
	Apomorphine										108		X
	Rotigotine (transdermal,								71				X

Drug classes	*Drugs*	*SRs with MA of RCTs*	*RCTs*	*SRs without MA*	*CS*	*CCS*	*CSS*	*OBS*	*NRIS*	*PVG-DB*	*Narrative reviews/ SmPC analysis*	*Associated with OH[1]:*	
												Bhanu et al. [10]	*Other study*
	add-on to Levodopa)												
	Amantadine (extended-release capsules)		55, 91								92	11/22/2024 2:26:00 PM	X
**ALPHA-2 AGONISTS**		10										X/	
	Dexmedeto-midine		50		40, 65, 98, 109						72		
***VOLTAGE-GATED SODIUM CHANNEL INHIBITORS**													
	Phenytoin							84			17		
	Carbamazepine										89		
	Lacosamide		60		95								
	Lidocaine		50										
**NITRATES**											11, 17, 72		X
	Glyceryl nitrate						27						
	Isosorbide dinitrate						96						
	Isosorbide mononitrate				112								
**ANTI-NEOPLASTICS AND IMMUNO-MODULATORS**		10										X/	
	mTOR-inhibitors									58			
	Trastuzumab							23					
	Pegteograstim							100					
	Tacrolimus									58			
*** (OTHER) VASODILATORS**													
	Iloprost (inhaled)								44				
	Prostacycline										72		
	Milrinone										72		
	Nitric oxide										72		
	Riociguat				35					52			
**SGLT-2 INHIBITORS**		10									90, 62	X	
	Canagliflozin										90		
**PDE-5 INHIBITORS**		10										X/	
	Tadalafil + Tamsulosin		79										X
**ANTIEMETICS**		10										X/	
	Ondansetron									28			

Drug classes	Drugs	SRs with MA of RCTs	RCTs	SRs without MA	CS	CCS	CSS	OBS	NRIS	PVG-DB	Narrative reviews/ SmPC analysis	Associated with OH[1]:	
												Bhanu et al. [10]	Other study
*NACHR ANTAGONISTS													
	Atracurium, Cisatracurium				31						72		
CORTICO-STEROIDS (EPIDURAL)		56											
ANTIANGINALS		10										X/	
GLP-1 AGONISTS		10										X/	
RADIOCONTRAST MEDIA					107								
SEDATIVES								76					
STATINS										25			
	Atorvastatin, Simvastatin									25			
*ACHEIS													
	Rivastigmine, Galantamine										89		
OTHER UNCLASSIFIED DRUGS													
	Propofol		21		109			76, 102	97	28	72		
	Finasteride		74								57		
	Dobutamine		42								72		
	Streptokinase				112		110						
	Ketamine						87	86					
	Lithium										17, 63		X
	Selegiline										11, 63		X
	Lanthanum carbonate	24											
	Etomidate		83										
	Rifapentine + Isoniazid		38										
	Inotersen				64								
	Conestat alfa				48								
	Levetiracetam (iv)				111								
	Regadenoson						53						
	Defibrotide								46				
	Warfarine									28			
	Clopidogrel									28			
	Remdesivir									75			
	Digoxin									28			
	Amiodarone										72		

												Associated with OH[1]:	
Drug classes	***Drugs***	***SRs with MA of RCTs***	***RCTs***	***SRs without MA***	***CS***	***CCS***	***CSS***	***OBS***	***NRIS***	***PVG-DB***	***Narrative reviews/ SmPC analysis***	***Bhanu et al. [10]***	***Other study***
	Buspirone										89		
	Clomethiazole										89		
	Hydroxyzine										89		
	N-Acetylcysteine										17		
	Memantine										11		X
	Methylphenidate										89		
	Modafinil										89		
	Pregabalin										89		
	Vancomycin										72		
	Vitamin K1 (parenteral)										105		

[1] Drugs or drug classes marked with a tick are reported to be associated with orthostatic hypotension either in the study by Bhanu et al. or in other studies. Drug classes marked with 'X/' were only mentioned in Bhanu et al. due to an insufficient number of studies available to conduct a comprehensive meta-analysis.

Journal: Archiv der Pharmazie

Identification of potentially causative drugs associated with hypotension: A scoping review

SUPPLEMENT S4: Study characteristics

Nurunnisa Sari [1,2], Ulrich Jaehde [3] and Anna Maria Wermund [3]*

[1] Institute for Medical Information Processing, Biometry and Epidemiology - IBE, LMU Munich, Germany

[2] Pettenkofer School of Public Health, Munich, Germany

[3] Department of Clinical Pharmacy, Institute of Pharmacy, University of Bonn, Germany

*Corresponding author. E-mail: a.wermund@uni-bonn.de

Table S1 *Included studies (ranked by year of publication)*

Reference	***First author***	***Title***	***Year of publication***	***Type of Evidence***	***Healthcare setting***	***Population characteristics***		
						**Country**	**Age**	**Special condition**
**83**	Hu B	Comparison of Remimazolam Tosilate and Etomidate on Hemodynamics in Cardiac Surgery: A Randomised Controlled Trial	2023	RCT	Hospital - During stay (surgery)	China	18 - 65	Scheduled for elective valve replacement surgery
**47**	Retnayyan A	Comparison of efficacy of tamsulosin, alfuzosin and silodosin in the management of benign prostatic hyperplasia	2023	Cohort study	Ambulatory	India	≥ 45	Lower urinary tract symptoms (LUTS) due to benign prostatic hyperplasia (BPH)
**64**	Luigetti M	Real-life experience with inotersen in hereditary transthyretin amyloidosis with late-onset phenotype: Data from an early-access program in Italy	2022	Cohort study	Ambulatory	Italy	59 - 82	Hereditary transthyretin amyloidosis with late-onset phenotype
**52**	Patel NM	A Pharmacovigilance Study of Adverse Drug Reactions Reported for Cardiovascular Disease Medications Approved Between 2012 and 2017 in the United States Food and Drug Administration Adverse Event Reporting System (FAERS) Database	2022	Retrospective evaluation of pharmacovigilance database	No information provided	USA	Mean age = 65.8 Older patient proportion 60%	-
**30**	Virnes RE	Opioids and Falls Risk in Older Adults: A Narrative Review	2022	Narrative review	No information provided	-	Older adults	-
**75**	Jung SY	Cardiovascular events and safety outcomes associated with remdesivir using a World Health Organization international pharmacovigilance database	2022	Retrospective evaluation of pharmacovigilance database	No information provided	Worldwide	-	COVID-19
**51**	Patel PC	Cardiovascular and Metabolic Adverse Reactions Associated with the Use of Antipsychotic Drugs: A Narrative Review	2022	Narrative review	No information provided	-	-	-
**104**	Butzner M	Adverse drug effects across patients with heart failure: a systematic review	2022	Systematic review without meta-analysis	No information provided	-	-	Heart failure
**86**	Groth CM	Multicenter Retrospective Review of Ketamine Use in the ICU	2022	Other observational studies	Hospital - During stay (ICU)	USA	39 - 65	-
**97**	De Melo IB	TRPA1 Polymorphisms Modify the Hypotensive Responses to Propofol with No Change in Nitrite or Nitrate Levels	2022	Non-randomised interventional study	Hospital - During stay (surgery)	Brazil	55 ± 14	Patients undergoing colonoscopy
**62**	Mascolo A	Safety profile of sodium glucose co-transporter 2 (SGLT2) inhibitors: A brief summary	2022	Narrative review	No information provided	-	-	
**27**	Yerni Kumar B	Medication Reconciliation Led by Clinical Pharmacist as an Effective Strategy in Preventing and Reducing Adverse Drug Reactions	2022	Cross-sectional study	Hospital - During stay (other ward)	India	> 25	-

Reference	*First author*	*Title*	*Year of publication*	*Type of Evidence*	*Healthcare setting*	*Population characteristics*		
						**Country**	**Age**	**Special condition**
**36**	Takuathung MN	Adverse Effects of Angiotensin-Converting Enzyme Inhibitors in Humans: A Systematic Review and Meta-Analysis of 378 Randomized Controlled Trials	2022	Systematic review of RCTs	No information provided	-	-	-
**78**	Jha P & Kumar D	Comparison of Adverse Drug Reactions among Antidepressant Drugs in the Outpatients Department of Psychiatry in a Tertiary Care Hospital of Eastern India	2022	Cohort study	Ambulatory	India	12 - 60	-
**111**	Alkazemi A	Safety of Intravenous Push Levetiracetam Compared to Intravenous Piggyback at a Tertiary Academic Medical Center: A Retrospective Analysis	2022	Cohort study	Hospital - During stay (multiple units)	USA	IVP group: 55.4 ± 16.4 IVPB group: 60.7 ± 15.3	-
**50**	Rekatsina M	Effects of Intravenous Dexmedetomidine Versus Lidocaine on Postoperative Pain, Analgesic Consumption and Functional Recovery After Abdominal Gynecological Surgery: A Randomized Placebo-controlled Double Blind Study	2021	RCT	Hospital - During stay (surgery)	Greece	30 - 70	Women, scheduled for abdominal hysterectomy or myomectomy
**40**	Sinnott J	The Use of Dexmedetomidine in the Emergency Department: A Cohort Study	2021	Cohort study	Hospital - Emergency Department	USA	≥ 18	-
**81**	Huang Y	Adverse Events of Sacubitril/Valsartan: A Meta-analysis of Randomized Controlled Trials	2021	Systematic review of RCTs	No information provided	-	-	-
**80**	Jaiswal SK	Preoperative Amlodipine Is Efficacious in Preventing Intraoperative HDI in Pheochromocytoma: Pilot RCT	2021	RCT	Hospital - During stay (surgery)	India	18 - 70	Pheochromocytoma and Paraganglioma (PPGL)
**32**	Van Poelgeest EP	Depression, antidepressants and fall risk: therapeutic dilemmas-a clinical review	2021	Narrative review	No information provided	-	-	-
**68**	Lee A & Shirley M	Remimazolam: A Review in Procedural Sedation	2021	Narrative review	Hospital - During stay (surgery)	-	-	Patients requiring endoscopies
**82**	Hunt NF	Safety of Intravenous Olanzapine Administration at a Tertiary Academic Medical Center	2021	Other observational studies	Hospital - During stay (multiple units)	USA	58-77	-
**33**	Thornby KA	A Systematic Review on the Use of Sacubitril/Valsartan Initiated Prior to Discharge in Hospitalized Patients With Heart Failure	2021	Systematic review without meta-analysis	Hospital - Discharge	-	-	Decompensated heart failure
**10**	Bhanu C	Drug-induced orthostatic hypotension: A systematic review and meta-analysis of randomised controlled trials	2021	Systematic review of RCTs	No information provided	-	≥ 18	-
**58**	Nguyen VN	Mtor inhibitors associated with higher cardiovascular adverse events-A large population database analysis	2021	Retrospective evaluation of pharmacovigilance database	No information provided	-	-	-

Reference	*First author*	*Title*	*Year of publication*	*Type of Evidence*	*Healthcare setting*	*Population characteristics*		
						**Country**	**Age**	**Special condition**
**65**	Liu S	Are dexmedetomidine and olanzapine suitable to control delirium in critically ill elderly patients? A retrospective cohort study	2021	Cohort study	Hospital - During stay (multiple units)	China	79.5 ± 5.4	-
**100**	Cheon J	Pegteograstim prophylaxis for chemotherapy-induced neutropenia and febrile neutropenia: a prospective, observational, postmarketing surveillance study in Korea	2021	Other observational studies	Hospital - During stay (other ward)	Korea	18 - 88	Administration after cancer-specific chemotherapy
**73**	Keller F	Arzneimittelnebenwirkungen – wie erkennen, wie vermeiden? [Adverse Drug Effects - how to detect, how to avoid?]	2021	Narrative review	No information provided	-	-	-
**7**	Sandoval T	Incident adverse drug reactions and their effect on the length of hospital stay in older inpatients	2021	Cohort study	Hospital - During stay (other ward)	Chile	≥ 60	-
**69**	Layek M	Angiotensin receptor neprilysin inhibitor in heart failure with reduced ejection fraction: Real-world experience from a safety perspective	2021	Non-randomised interventional study	Inpatient + Outpatient	-	54.3 ± 12.4	Acute decompensated heart failure & chronic compensated heart failure
**54**	Otero MJ	Utility of a trigger tool (TRIGGER-CHRON) to detect adverse events associated with high-alert medications in patients with multimorbidity	2020	Other observational studies	Hospital - Discharge	Spain	median = 84 (65 - 99)	
**109**	Benken S	Hemodynamic Effects of Propofol and Dexmedetomidine in Septic Patients Without Shock	2020	Cohort study	Hospital - During stay (ICU)	-	≥ 18	Non-vasopressor-dependent sepsis
**49**	Poyurovsky & Weizman	Treatment of Antipsychotic-Induced Akathisia: Role of Serotonin 5-HT2a Receptor Antagonists	2020	Narrative review	No information provided	-	-	Parkinson's disease
**11**	Rivasi G	Drug-Related Orthostatic Hypotension: Beyond Anti-Hypertensive Medications	2020	Narrative review	No information provided	-	-	-
**56**	Oliveira CB	Epidural corticosteroid injections for lumbosacral radicular pain	2020	Systematic review of RCTs	No information provided	-	mean = 37.3 - 52.8	-
**35**	Tanabe N	Safety and effectiveness of riociguat for chronic thromboembolic pulmonary hypertension in real-world clinical practice: interim data from post-marketing surveillance in Japan	2020	Cohort study	No information provided	Japan	66,1 ± 12,7	-
**88**	Golikhatir I	The Efficacy and Safety of Prochlorperazine in Patients With Acute Migraine: A Systematic Review and Meta-Analysis	2019	Systematic review of RCTs	Hospital - Emergency Department	-	>17	Acute migraine
**90**	Fitchett D	A safety update on sodium glucose co-transporter 2 inhibitors	2019	Narrative review	No information provided	-	-	-

Reference	*First author*	*Title*	*Year of publication*	*Type of Evidence*	*Healthcare setting*	*Population characteristics*		
						**Country**	**Age**	**Special condition**
**43**	Sarocchi M	Ivabradine in Cancer Treatment-Related Left Ventricular Dysfunction	2019	Cohort study	Ambulatory	Italy	median = 54	Cancer treatment-related left ventricular dysfunction
**63**	Manolis TA	Cardiovascular Safety of Psychiatric Agents: A Cautionary Tale	2019	Narrative review	No information provided	-	-	
**20**	Zullo AR	Patient-Important Adverse Events of beta-blockers in Frail Older Adults after Acute Myocardial Infarction	2019	Cohort study	No information provided	USA	≥ 65	Nursing home residents, hospitalized for AMI
**85**	Harvey PD	Blonanserin vs risperidone in Japanese patients with schizophrenia: A post hoc analysis of a phase 3, 8-week, multicenter, double-blind, randomized controlled study	2019	RCT	No information provided	Japan	≥ 15	Schizophrenia
**95**	Davidson KE	Safety and Efficiency of Intravenous Push Lacosamide Administration	2018	Cohort study	Hospital - During stay (multiple units)	USA	49 - 69	-
**66**	Lee YK	Antidepressants-related cardiovascular adverse events using the adverse event reporting system	2018	Retrospective evaluation of pharmacovigilance database	No information provided	USA & Korea	≥ 19	-
**101**	Channell JS	Toxicity of tapentadol: a systematic review	2018	Systematic review without meta-analysis	No information provided	-	-	-
**91**	Elmer LW	Pooled Analyses of Phase III Studies of ADS-5102 (Amantadine) Extended-Release Capsules for Dyskinesia in Parkinson's Disease	2018	RCT	Ambulatory	North America & Western Europe	mean = 64	-
**92**	Elkurd MT	The role of extended-release amantadine for the treatment of dyskinesia in Parkinson's disease patients	2018	Narrative review	No information provided	-	-	-
**105**	Britt RB	Characterizing the Severe Reactions of Parenteral Vitamin K1	2018	Narrative review	No information provided	-	28 - 92	-
**19**	Han SE	Safety and efficacy of fimasartan with essential hypertension patients in real world clinical practice: data from a post marketing surveillance in Korea	2018	Cohort study	Hospital - During stay	Korea	63,8 ± 12,1	Hypertensive patients
**87**	Groth CM	Current practices and safety of medication use during rapid sequence intubation	2018	Cross-sectional study	Hospital - During stay (multiple units)	USA	58 ± 22	-
**110**	Aslanabadi N	The Streptokinase Therapy Complications and its Associated Risk Factors in Patients with Acute ST Elevation Myocardial Infarction	2018	Cross-sectional study	Hospital - During stay	Iran	≥ 18	Acute ST elevation myocardial infarction
**112**	Acharya T	Pharmacovigilance study of patients receiving treatment at medical ICU of C. U. Shah medical college & hospital, Surendranagar	2018	Cohort study	Hospital - During stay (ICU)	India	No restriction	-

Reference	*First author*	*Title*	*Year of publication*	*Type of Evidence*	*Healthcare setting*	*Population characteristics*		
						**Country**	**Age**	**Special condition**
**84**	Höhne J	The risk of hypotension and seizures in patients receiving prophylactic anti-epileptic drugs for supratentorial craniotomy	2018	Other observational studies	Hospital - During stay (surgery)	Germany	mean = 50	Primary or secondary supratentorial brain tumors
**55**	Oertel W	Randomized, Placebo-Controlled Trial of ADS-5102 (Amantadine) Extended-Release Capsules for Levodopa-Induced Dyskinesia in Parkinson's Disease (EASE LID 3)	2017	RCT	Inpatient + Outpatient	USA, Ger-many, France, Spain, and Austria	30 - 85	Parkinson's disease
**60**	Misra UK	Comparison of lacosamide versus sodium valproate in status epilepticus: A pilot study	2017	RCT	Hospital - During stay	India	18 - 90	Consecutive patients with convulsive Status Epilepticus or subtle convulsive Status Epilepticus
**89**	Freudenmann RW	Arterial Hyper- and Hypotension associated with psychiatric medications: a risk assessment based on the summaries of product characteristics (SmPCs)	2017	SmPC analysis	No information provided	-	-	-
**31**	VanderWeide LA	The Incidence of hypotension with continuous infusion atracurium compared to cisatracurium in the Intensive Care Unit	2017	Cohort study	Hospital - During stay (ICU)	-	18 - 89	-
**67**	Lee HY	Propacetamol poses a potential harm of adverse hypotension in male and older patients	2017	Retrospective evaluation of pharmacovigilance database	No information provided	Korea	53,9 ± 25,8	Male sex, concomitant medications identified as significant risk factors
**46**	Richardson PG	Defibrotide for Patients with Hepatic Veno-Occlusive Disease/Sinusoidal Obstruction Syndrome: Interim Results from a Treatment IND Study	2017	Non-randomised interventional study	Hospital - During stay	USA	median = 42	Veno-occlusive disease/sinosoidal obstruction syndrome
**25**	You T	Effect of statins on blood pressure: Analysis on adverse events released by FDA	2017	Retrospective evaluation of pharmacovigilance database	No information provided	USA	-	-
**96**	De Figueiredo TP	Factors associated with adverse drug reactions in older inpatients in teaching hospital	2017	Cross-sectional study	Hospital - During stay (other ward)	Brazil	≥ 60	-
**107**	Blackwell RH	Incidence of Adverse Contrast Reaction Following Nonintravenous Urinary Tract Imaging	2016	Cohort study	Hospital - During stay	USA	70,7 ± 15,4	Patients who underwent urologic procedure
**108**	Bhidayasiri R	Practical management of adverse events related to apomorphine therapy	2016	Narrative review	No information provided	-	-	Parkinson's disease
**53**	Pape M	Safety and tolerability of regadenoson for myocardial perfusion imaging - first Danish experience	2016	Cross-sectional study	Inpatient + Outpatient	Denmark	63 ± 12,4	Suspected or known coronary artery disease
**28**	Wang K	Opportunities for Web-based Drug Repositioning: Searching for Potential Antihypertensive Agents with Hypotension Adverse Events	2016	Retrospective evaluation of	No information provided	USA	-	-

Reference	*First author*	*Title*	*Year of publication*	*Type of Evidence*	*Healthcare setting*	*Population characteristics*		
						**Country**	**Age**	**Special condition**
				pharmacovigilance database				
**93**	El-Saifi N	Quetiapine safety in older adults: a systematic literature review	2016	Systematic review without meta-analysis	No information provided	-	≥ 65	-
**74**	Kaplan SA	Time Course of Incident Adverse Experiences Associated with Doxazosin, Finasteride and Combination Therapy in Men with Benign Prostatic Hyperplasia: The MTOPS Trial	2016	RCT	No information provided	-	≥ 50	Benign prostatic hyperplasia
**29**	Viscuisi ER	A comparison of opioid-related adverse events with fentanyl iontophoretic transdermal system versus morphine intravenous patient-controlled analgesia in acute postoperative pain	2016	Narrative review	No information provided	-	mean = 50.2 - 62.9	Acute postoperative pain
**44**	Saji T	Efficacy and safety of inhaled iloprost in Japanese patients with pulmonary arterial hypertension - Insights from the IBUKI and AIR Studies	2016	Non-randomised interventional study	Ambulatory	Japan	18 - 75	Pulmonary arterial hypertension
**79**	Jin BH	Pharmacokinetics and safety profiles of tadalafil/tamsulosin HCL fixed-dose combination capsule under fasted and fed condition in healthy volunteers	2016	RCT	No information provided	Korea	mean = 25	-
**22**	Zhang X	Efficacy and Safety of Vasopressin Receptor Antagonists for Euvolemic or Hypervolemic Hyponatremia: A Meta-Analysis	2016	Systematic review of RCTs	No information provided	-	≥ 18	-
**45**	Ryu YW	Comparison of tamsulosin plus serenoa repens with tamsulosin in the treatment of benign prostatic hyperplasia in Korean men: 1-year randomized open label study	2015	RCT	Hospital - During stay (other ward)	Korea	50 - 80	-
**34**	Tang HN	Terazosin versus alfuzosin in treatment of acute urinary retention in patients with benign prostatic hypertrophy	2015	Case-control study	Hospital - Emergency Department	Hong Kong	72 ± 18.1	Acute urinary retention in BPH patients
**38**	Sterling TR	Flu-like and Other Systemic Drug Reactions Among Persons Receiving Weekly Rifapentine Plus Isoniazid or Daily Isoniazid for Treatment of Latent Tuberculosis Infection in the PREVENT Tuberculosis Study	2015	RCT	Ambulatory	USA, Canada, Brazil, Spain	≥ 12	High risk for tuberculosis
**102**	Campbell SG	Emergency procedural sedation with propofol in older teenagers any cause for concern?	2015	Other observational studies	Hospital - Emergency Department	Canada	mean = 43 (adult population); mean = 77 (senior population)	-
**94**	El Said NO	Alfuzosin treatment improves the rate and time for stone expulsion in patients with distal uretral stones: a prospective randomized controlled study	2015	RCT	Ambulatory	Egypt	≥ 18	Uncomplicated radio-opaque stones
**72**	Kim JM	Rotigotine transdermal system as add-on to oral dopamine agonist in advanced Parkinson's disease: An open-label study	2015	Non-randomised interventional study	Ambulatory	South Korea, Malaysia, Taiwan, Australia	30 - 80	Advanced Parkinson’s disease

Reference	*First author*	*Title*	*Year of publication*	*Type of Evidence*	*Healthcare setting*	*Population characteristics*		
						**Country**	**Age**	**Special condition**
						and Singapore		
**23**	Zhang M	Herceptin as a single agent in the treatment of patients with metastatic breast cancer	2015	Other observational studies	No information provided	-	25 - 72	Breast cancer
**39**	Spindelegger CJ	Cardiovascular adverse reactions during antidepressant treatment: A drug surveillance report of German-speaking countries between 1993 and 2010	2015	Retrospective evaluation of pharmacovigilance database	Hospital - During stay	Germany, Austria, Switzerland	-	Psychiatric inpatients
**42**	Shehata M	Accelerated dobutamine stress testing: Feasibility and safety in patients with moderate aortic stenosis	2014	RCT	No information provided	Egypt	62.29 ± 9.8	Calcific moderate valvular aortic stenosis
**76**	Kane-Gill SL	A multicenter study of the point prevalence of drug-induced hypotension in the ICU	2014	Other observational studies	Hospital - During stay (ICU)	USA, Canada, Singapore	59.9 ± 16	-
**57**	Oelke M	Cardiovascular and ocular safety of α1-adrenoceptor antagonists in the treatment of male lower urinary tract symptoms	2014	Narrative review	No information provided	-	-	-
**106**	Boyd K	alpha-Adrenergic Blockers for the Treatment of Lower-Urinary-Tract Symptoms and Dysfunction in Women	2014	Narrative review	No information provided	-	-	-
**77**	Joel JJ	Evaluation of adverse drug reactions associated with the psychotropic drugs in the management of patients with schizophrenia	2014	Cohort study	Hospital - During stay (other ward)	India	18 - 60	Schizophrenia
**98**	Crispo AL	Comparison of clinical outcomes in nonintubated patients with severe alcohol withdrawal syndrome treated with continuous-infusion sedatives: Dexmedetomidine versus benzodiazepines	2014	Cohort study	Hospital - During stay (multiple units)	USA	48 ± 11 (BZD group) 55 ± 13 (DEX group)	Severe alcohol withdrawal syndrome
**59**	Nair B	Making clozapine safer: Current perspectives on improving its tolerability	2014	Narrative review	No information provided	-	-	-
**61**	Michl J	A multivariate approach linking reported side effects of clinical antidepressant and antipsychotic trials to in vitro binding affinities	2014	Narrative review	No information provided	-	-	-
**41**	Shimamoto K	Nifedipine controlled-release 40 mg b.i.d. in Japanese patients with essential hypertension who responded insufficiently to nifedipine controlled-release 40 mg q.d.: A phase III, randomized, double-blind and parallel-group study	2014	RCT	Ambulatory	Japan	33 – 85	-
**21**	Zhou Y	Midazolam and propofol used alone or sequentially for long-term sedation in critically ill, mechanically ventilated patients: a prospective, randomized study	2014	RCT	Hospital - During stay (ICU)	China	54.8 ± 13.6	-

Reference	*First author*	*Title*	*Year of publication*	*Type of Evidence*	*Healthcare setting*	*Population characteristics*		
						**Country**	**Age**	**Special condition**
**24**	Zhang C	Efficacy and safety of lanthanum carbonate on chronic kidney disease-mineral and bone disorder in dialysis patients: a systematic review	2013	Systematic review of RCTs	Hospital - During stay	-	-	Chronic kidney disease - mineraland bone disorder (CKD-MBD)
**72**	Kennelly C	Drug-induced cardiovascular adverse events in the intensive care unit	2013	Narrative review	Hospital - During stay (ICU)	-	-	-
**37**	Stuebner E	Twenty-four hour non-invasive ambulatory blood pressure and heart rate monitoring in Parkinson's Disease	2013	Narrative review	No information provided	-	-	Parkinson's disease
**103**	Calver L	A prospective study of high dose sedation for rapid tranquilisation of acute behavioural disturbance in an acute mental health unit	2013	Cohort study	Hospital - During stay (other ward)	USA	median = 40; range = 15 - 80	Acute behavioural disturbance
**48**	Reshef A	Recombinant human C1 inhibitor for the prophylaxis of hereditary angioedema attacks: A pilot study	2013	Cohort study	Ambulatory	Romania, Israel, Poland	37.9 ± 13.4	C1 inhibitor deficiency, History of Hereditary angioedema (HAE)
**99**	Cox ZL	Adverse drug events during AKI and its recovery	2013	Cohort study	Hospital - Admission	USA	60 ± 16	Elevated serum creatinine; acute kidney injury (AKI)
**26**	Yoshimura K	A survey of the FAERS database concerning the adverse event profiles of α1-adrenoreceptor blockers for lower urinary tract symptoms	2013	Retrospective evaluation of pharmacovigilance database	No information provided	-	-	Lower urinary tract symptoms
**70**	Kutlu R	Comparison of the safety and efficacy of ivabradine and nebivolol mono- and combination therapies in the treatment of stable angina pectoris patients with left ventricular dysfunction	2013	Non-randomised interventional study	Ambulatory	-	61 ± 5.1	Stable angina pectoris
